# UAV-Based Person Detection with Frame and Event Sensors: A Model–Platform Case Study on Jetson Orin Nano and BrainChip AKD1000 Neuromorphic Hardware

**DOI:** 10.3390/s26217034

**Published:** 2026-11-06

**Authors:** Fernando Palacios, Ana Redondo Rodriguez, Hélder Rodríguez, Salvador Domínguez-Durante, Daniel Sanz-Merodio, Miguel López

**Affiliations:** ARQUIMEA Robotics, Arquimea Research Center, 38320 La Laguna, Spain; aredondo@arquimea.com (A.R.R.); hrodriguez@arquimea.com (H.R.); sdominguez@arquimea.com (S.D.-D.); dsanz@arquimea.com (D.S.-M.); mlopez@arquimea.com (M.L.)

**Keywords:** neuromorphic computing, performance evaluation and benchmarking, aerial systems: applications, object detection, spiking neural network (SNN)

## Abstract

Detecting people from unmanned aerial vehicles (UAVs) in coastal environments is challenging because targets are often small, distant, and observed against complex backgrounds. This case study presents an application-driven system-level comparison of frame- and event-based sensing vision systems. We developed a purpose-built multimodal UAV dataset of beach users, bathers, and surfers using frame and event-based cameras. Frame images and events were evaluated using feasible standard and converted spiking YOLOv2-derived configurations on a Jetson Orin Nano and a BrainChip AKD1000 neuromorphic processor. The experimental setup serves as a challenging stress test to expose limitations and relative behavior across configurations. The evaluation covers end-to-end processing performance and energy and sensor power. Frame-based YOLOv2 achieved the highest detection performance, with an F1-score of 0.215 and an mAP50 of 0.212. The best spiking configuration, sYOLOv2 with event input, achieved an F1-score of 0.172 and an mAP50 of 0.114. Frame-based YOLOv2 on the Jetson GPU also achieved the lowest end-to-end processing energy cost (2886.6 mJ per sample) and highest throughput (2.011 samples/s). At the component level, the event camera reduced operating power by 82.1% relative to the frame camera, while the AKD1000 consumed approximately 1.3 W during backend execution. However, these component-level savings did not yield an end-to-end energy advantage because of host-processing overhead and lower throughput. The results highlight the need to assess sensing and complete-pipeline costs when evaluating the efficiency of neuromorphic embedded vision systems.

## 1. Introduction

Unmanned aerial vehicles (UAVs) provide a flexible platform for monitoring coastal areas and can support the early identification of people exposed to hazardous conditions. Previous research has demonstrated the feasibility of UAV-based computer vision for detecting people in coastal zones [1] and open water [2]. Nevertheless, reliable onboard detection remains challenging because people observed from aerial coastal viewpoints are often small and distant, while their appearance varies with activity, viewing angle, illumination, reflections, sea state, and partial occlusion. Moreover, UAV applications impose restrictions on payload, computational resources, thermal dissipation, communication bandwidth, and battery capacity [3]. Consequently, their practical suitability depends not only on detection performance but also on the power and processing requirements of the acquisition and computing subsystems.

Neuromorphic technology, designed to emulate biological neural behavior in electronic systems, has emerged as a promising paradigm for achieving low-power, high-throughput processing in machine learning applications. This is particularly relevant in robotics, where efficient computation is critical for real-time decision making in resource-constrained environments [4].

Event cameras and neuromorphic processors offer potentially useful characteristics for resource-constrained perception. Event cameras asynchronously report brightness changes rather than recording complete images at fixed intervals, providing high temporal resolution, high dynamic range, and reduced motion blur while generating sparse data when scene activity is limited [5]. Neuromorphic processors are designed to exploit sparse, event-driven computation and can execute compatible spiking neural networks using low-precision representations [6,7]. However, low sensor or accelerator power does not necessarily imply low system-level energy, because event accumulation, input conversion, host–device communication, model execution, and output decoding may introduce substantial costs outside the sensor or neuromorphic accelerator.

Accordingly, this study investigates the practical trade-offs among feasible sensor, input-representation, model, and computing-platform configurations for UAV-based person detection in coastal environments. The AKD1000 was selected as the neuromorphic target because it provided a commercially available physical accelerator that could be directly integrated with a high-performance general-purpose embedded platform such as the Jetson Orin Nano, while providing an established workflow for converting Artificial Neural Networks (ANNs) into Spiking Neural Networks (SNNs) and deploying the converted models on the neuromorphic hardware. Specifically, this case study addresses the following research question: what performance and system-level efficiency trade-offs arise when a pipeline for detecting people from UAVs in coastal environments is deployed on a Jetson Orin Nano with an AKD1000 neuromorphic accelerator?

To support this objective, we have developed an artificial vision system that integrates both classical and neuromorphic components across multiple stages, from sensing to computation. Our system captures data in a challenging outdoor environment using UAV-mounted sensors, including conventional frame cameras and event-based Dynamic Vision Sensors (DVS) [8,9]. We deployed YOLOv2 detection neural network models, both classical and spiking, on a Jetson Orin Nano computing platform and measured the performance on CPU, GPU, and an AKD1000 neuromorphic processor [10]. The experimental setup is intended as a stress test to expose limitations and relative behaviors across the evaluated configurations rather than as a deployment-ready coastal-safety system.

The main contribution of this case study is a system-level evaluation of conventional frame-based and neuromorphic event-based sensing and computing configurations for coastal-safety detection, encompassing detection performance across frame images and accumulated events; feasible standard, quantized, and converted spiking YOLOv2-derived model–platform configurations; and resource metrics from sensor active/standby power to preprocessing, backend execution, postprocessing, throughput, power, and end-to-end energy. This scope explicitly includes sensor-level consumption, which is commonly omitted from system-level neuromorphic benchmarking studies.

## 2. Related Work

### 2.1. UAV-Based Human Detection in Coastal and Maritime Environments

Unmanned aerial vehicles have become relevant platforms for visual surveillance and search-and-rescue operations because they can rapidly cover large or inaccessible areas and provide aerial observations at different altitudes and viewing angles. Previous studies have applied convolutional object detectors to human detection in nadir and oblique UAV imagery, showing that detection performance is strongly influenced by flight altitude, ground sampling distance, camera orientation, environmental conditions, and the availability of representative training data [11,12].

Within maritime environments, the SeaDronesSee benchmark [12] introduced a large-scale dataset for detecting and tracking humans in open water from conventional UAV imagery and provided baseline evaluations for several object-detection methods. Subsequent work has developed scale-adaptive architectures specifically for open-water search and rescue, such as YoloOW [2], which addresses the substantial variation in target size found in UAV imagery. In the coastal-monitoring domain, Da-Silva-Gomez et al. [1] evaluated UAV-based computer-vision methods for hazardous-area segmentation and for detecting beach users, bathers, swimmers, and surfers. Together, these studies demonstrate the relevance of UAV-based human detection for coastal safety and maritime rescue.

Objects observed from UAVs frequently occupy only a small number of pixels and consequently contain limited appearance and contextual information. Their detection is further complicated by scale variation, occlusion, complex backgrounds, image degradation, and class imbalance [13]. These difficulties are particularly relevant to human detection from elevated viewpoints and to maritime imagery, where people in the water may be visually similar to waves, foam, reflections, or other background structures [2,12]. However, the sensing systems considered in this literature are predominantly based on conventional frame cameras.

### 2.2. Event-Based Vision for Object Detection

Event cameras asynchronously report pixel-level brightness changes instead of acquiring complete images at a fixed frame rate. Their high temporal resolution, high dynamic range, sparse output, and reduced sensitivity to motion blur have motivated extensive research in robotics and computer vision [5]. These properties have supported applications such as visual–inertial odometry and simultaneous localization and mapping under high-speed motion and difficult illumination conditions [14].

Event streams can be processed directly using asynchronous or spatiotemporal algorithms, or they can be aggregated into frame-like images, histograms, voxel grids, or other tensor representations compatible with conventional neural networks. Gehrig et al.  [15] presented a general framework for learning grid-based representations from asynchronous events and showed how many existing event representations can be expressed within a common formulation. The selection of a representation determines how much temporal information and sparsity are retained and therefore affects both detection performance and computational requirements.

For object detection, Perot et al. [16] introduced a high-resolution event-camera dataset and a recurrent architecture capable of detecting vehicles and pedestrians directly from event data without reconstructing intensity images. Gehrig and Scaramuzza [17] subsequently proposed recurrent vision transformers that combine spatial processing with temporal state for low-latency event-based detection. More recently, Chen et al. [18] introduced an event-based tiny-object benchmark for UAV detection and demonstrated the relevance of spatiotemporal continuity when targets occupy only a few pixels. Nevertheless, these representative event-based detection studies primarily address automotive or anti-UAV scenarios rather than the detection of people in coastal environments from a drone-mounted event camera.

### 2.3. Neuromorphic Hardware and System-Level Benchmarking

Neuromorphic processors are designed to support sparse and event-driven computation, frequently combining low-precision representations with architectures tailored to spiking neural networks [19]. Their potential efficiency depends not only on the neural model but also on the mapping procedure, numerical precision, data representation, communication interfaces, and the amount of processing performed outside the accelerator. Consequently, chip-level power or operation counts alone do not necessarily represent the energy required by a complete application.

Several alternative hardware approaches have demonstrated competitive performance or energy efficiency for specific SNN and event-driven workloads. Intel Loihi 2 [20] provides a programmable digital neuromorphic architecture and has demonstrated advantages over conventional hardware for sparse-computing workloads; for example, Parpart et al. [6] reported at least 34% faster execution and an order-of-magnitude improvement in energy efficiency over the evaluated CPU and GPU implementations for the Locally Competitive Algorithm under sufficiently large sparsity penalties, while maintaining comparable reconstruction quality. SynSense follows a more tightly integrated sensing–computing approach; its Speck platform combines a Dynamic Vision Sensor with fully asynchronous SNN processing in a single system-on-chip and has demonstrated milliwatt-level operation in event-based vision workloads [21]. FPGA-based SNN accelerators constitute another alternative, allowing processing architectures, memory organization, and parallelism to be tailored to specific workloads. For example, López-Asunción and Ituero [22] reported an edge-oriented FPGA implementation operating at 348 mW, while hardware-aware frameworks such as SpikeExplorer enable the joint exploration of accuracy, latency, power, and resource utilization for FPGA-based SNN implementations [23]. These results were obtained using different models, datasets, hardware configurations, and measurement boundaries and therefore provide technological context rather than a direct quantitative comparison among platforms. Moreover, these approaches follow different deployment models: Loihi 2 is primarily a research platform, Speck tightly integrates event-based sensing and neuromorphic processing, and FPGA-based solutions generally require hardware-specific design and mapping. Consequently, adopting these alternatives would imply substantially different hardware integration and model-deployment workflows from the host-connected accelerator and ANN-to-SNN conversion approach considered in this study.

At the benchmarking level, NeuroBench defines complementary algorithm and system tracks for evaluating neuromorphic solutions [7]. Its system track explicitly considers deployed execution and includes task-level measures such as latency, throughput, power, and energy, with preprocessing and postprocessing included within the measurement boundary. Gamage et al. [24] further compared frame- and event-based ANN and SNN object-detection configurations across neuromorphic and conventional edge-computing platforms, including a Jetson Orin Nano. However, none of these studies have included power consumption measurements of the sensors used to collect the data.

### 2.4. BrainChip Akida Platform

BrainChip’s Akida platform comprises physical edge-AI processors and processor-IP implementations based on its fully digital, event-based neuromorphic architecture. The AKD1000 employed in this study implements the first-generation Akida architecture and integrates 20 Neural Processing Cores together with standard interfaces including PCIe, enabling its use as a physical accelerator connected to a host computing platform [10]. This host–accelerator configuration is particularly relevant for heterogeneous edge-computing systems in which general-purpose processing and neuromorphic inference are combined. BrainChip has subsequently introduced the AKD1500, also based on the Akida 1.0 architecture and targeted at lower-power, more compact edge deployments. The AKD1500 has recently become commercially available in compact edge-oriented form factors [25].

BrainChip has also introduced Akida 2 as its second-generation processor-IP architecture. Akida 2 extends the first-generation architecture with additional numerical-precision and spatiotemporal-processing capabilities, including support for Temporal Event-Based Neural Networks (TENNs) [26]. At present, Akida 2 is available as processor IP and can be evaluated through remotely hosted Akida Cloud resources or FPGA-based development platforms intended for model evaluation, validation, and system integration, rather than through a production edge accelerator directly equivalent to the AKD1000 [27].

The BrainChip AKD1000 has been evaluated in several application domains. Le et al. [28] deployed AkidaNet for edge-based crop and weed recognition, while [29] investigated low-power cloud-cover detection for onboard satellite processing. Lutes et al. [30] used the AKD1000 for individualized braking-intent detection based on electroencephalographic signals. These studies demonstrate the applicability of the processor for classification and edge-inference tasks.

## 3. Materials and Methods

### 3.1. Hardware Setup

#### 3.1.1. Cameras

As part of the system-level approach, a crucial component of the system is the type of input data used by the networks. Therefore, image sensors from different technologies were selected: traditional frame-based sensors and event-based sensors. This choice enhances the evaluation of both sensing technologies within the considered case study. As the classical vision system, we employed the acquisition setup previously used in  [1]. For this study, we only considered a frame-based camera (FLIR Blackfly S, Teledyne FLIR Integrated Imaging Solutions, Richmond, BC, Canada) with a resolution of 2448 × 2048 pixels (although the resolution employed was 1224 × 1024 after pixel binning) and an event-based camera (Prophesee EVK4 HD sensor, Prophesee, Paris, France) with a resolution of 1280 × 720 pixels. The two cameras were temporally coordinated at the software acquisition level. The FLIR camera operated in continuous acquisition mode, while the event camera continuously acquired asynchronous events. A software synchronization mechanism coordinated both acquisition streams, generating approximately one accumulated event image for each FLIR image acquisition cycle, with an effective integration interval of approximately 90 ms. Each event image contained the events accumulated by the event-frame generator up to the timestamp of the latest processed event batch. In parallel with the generation of these event images, the complete asynchronous event stream was recorded in raw format, preserving the original event data for subsequent processing.

#### 3.1.2. Computing Platforms

The system leverages a heterogeneous computing architecture mounted on a Jetson Orin Nano platform (NVIDIA Corporation, Santa Clara, CA, USA), combining classical and neuromorphic processing units.

- CPU: A 6-core Arm® Cortex®-A78AE v8.2 64-bit CPU with 1.5 MB L2 and 4 MB L3 cache, operating at 1.7 GHz, providing general-purpose processing for control and data management tasks.
- GPU: A 1024-core NVIDIA Ampere architecture GPU with 32 Tensor Cores, running at 1020 MHz, optimized for parallel processing and acceleration of deep learning workloads (e.g., CNNs for detection and segmentation).
- Brainchip AKD1000 (BrainChip Inc., Laguna Hills, CA, USA): This chip is a digital, event-based accelerator based on the Akida 1.0 neuromorphic processor architecture. In the Akida 1.0 workflow, quantized models are converted for execution on the Akida platform. Its neural processing units process non-zero values as events and combine computation with local memory resources [31,32]. This processor was connected to the Jetson Orin Nano platform through Peripheral Component Interconnect Express (PCIe), using an ADT-Link R43DF PCIe riser adapter.

#### 3.1.3. UAV for Data Acquisition

The cameras and the Jetson Orin Nano platform were mounted on a DJI Matrice 350 RTK UAV (SZ DJI Technology Co., Ltd., Shenzhen, China) for data acquisition (Figure 1). During acquisition, the Jetson platform was employed to control both cameras in order to acquire synchronized data and to store frames and raw event data.

### 3.2. Dataset

The dataset used for this research was collected in diverse coastal environments, encompassing four beaches at different times of the day and under varying meteorological conditions to ensure robust training capabilities (Figure 2). The beaches selected were located at Tenerife (Canary Islands, Spain), and they provided a comprehensive representation of the coastal morphologies. Those beaches were: Las Teresitas (lat. 28°30′30.7″ N; long. 16°11′34.2″ W), El Socorro (lat. 28°23′38.3″ N; long. 16°36′10.0″ W), Jardín (lat. 28°24′44.4″ N, 16°33′31.3″ W) and El Abrigo (lat. 28°13′16.7″ N, long. 16°24′47.9″ W).

The dataset consisted of 44 raw event-based and frame-based video recordings, each approximately 10 s in duration. The videos were recorded with the UAV hovering at a horizontal distance between 150 and 200 m from the shoreline and at an altitude of approximately 20 m. For the task, we focus on detecting three distinct classes: beach users, surfers, and bathers. These categories were chosen to evaluate the networks’ ability to distinguish between visually similar but behaviorally different subjects. Not all classes were observed at all beaches or in every recording (e.g., surfers appeared only at El Socorro and not in every recording from that beach), so the data split strategy guaranteed that all three classes (beach users, surfers, bathers) were present in all splits (train, validation and test), that the proportions of each class in each split were similar to the overall class distribution in the dataset, that each video (and thus all of its frames and event sequences) appeared in exactly one split (in order to avoid data leakage), and that all four beaches were represented in every split with recordings spanning different times and days. The number of objects and their distribution are specified in Table 1 demonstrating a balanced representation across all splits, giving the following percentage for training, validation and testing: 70.6%–12.5%–16.9%.

### 3.3. Data Types

To analyze the behavior of different network architectures and computing configurations across various data types, we selected a diverse dataset encompassing multiple modalities. Given that the input data was visual, we employed three distinct representations: frame-based data, event-based images captured by DVS cameras, and temporally structured tensors. Frame data consisted of single-channel grayscale images with a spatial resolution of 1224 × 1024 pixels after sensor binning. As described in Section 3.1.1, the event-based images were formed online during acquisition from accumulated events. For training and processing, all events were encoded as 255 and background pixels as 0, producing a polarity-agnostic, binary event image. These event images consisted of a single channel with the native spatial resolution of the event sensor (1280 × 720 pixels). The tensor representation was instead generated from the asynchronously recorded raw event stream. The tensor approach involved discretizing raw event data into temporal steps, effectively transforming asynchronous event streams into structured time-based representations as shown in Figure 3a–d. A total of eight temporal steps were used, a choice motivated by the number of temporal steps employed in SNN implementation of YOLOv4 work [33], in which four and eight steps were evaluated for object detection and with a slight improvement reported at eight steps, with a mean Average Precision (mAP) of 53.25%. Each tensor represented approximately the same temporal interval as an accumulated event image, but preserved greater temporal granularity by dividing this interval into eight evenly spaced temporal steps, with an integration window of approximately 11.25 ms per step. Each temporal step had the native 1280 × 720 spatial resolution of the event sensor, with a value of 1 indicating that at least one event occurred at that pixel during the corresponding temporal interval and 0 indicating no event. The eight temporal steps were represented as separate channels, resulting in a 1280 × 720 × 8 tensor representation. For model processing, all three representations were subsequently divided into spatial patches following the common sliding-window procedure described in Section 3.7, resulting in 224 × 224 single-channel inputs for frame and event data and 224 × 224 × 8 inputs for tensor data. A sample of frame-based and event-based views are displayed on Figure 3e,f. This diverse selection allowed for a comprehensive evaluation of the models’ adaptability to different data formats and temporal dynamics throughout different hardware platforms.

### 3.4. Network Architectures

The detection task was chosen given its intrinsic complexity, which presents significant challenges in both localization and classification, thereby providing a rigorous benchmark for evaluating model performance.

The architecture was initially selected on the basis of YOLOv4-tiny [34]. This model was chosen due to the availability of its neuromorphic counterpart, as described in [33]. However, due to the model’s complexity, it could only be deployed on CPU- and GPU-based platforms, and was therefore discarded. Consequently, an alternative detection model was deployed based on YOLOv2 [35], an earlier version. This version employed AkidaNet [28] as the backbone, which was compatible with the available neuromorphic hardware. The AkidaNet backbone was configured with a width multiplier (referred to as “alpha”) of 0.5, which reduced the number of filters in each convolutional layer by half relative to the original AkidaNet configuration [36]. This value was selected because configurations with larger width multipliers could not be fully mapped onto the AKD1000 hardware. For the YOLOv2 detection head, five anchors were employed [37].

The YOLOv2 network was quantized and converted to Spiking Neural Network employing the CNN2SNN toolkit from the Akida Machine Learning framework [38], which converted the model to an Akida V1 SNN model that could be deployed on AKD1000. Although there is a second version of the Akida framework, it is not compatible with the AKD1000 chip. The quantizer provided by the CNN2SNN toolkit replaces the neural Keras layers (Conv2D, SeparableConv2D and Dense) and the ReLU layers with custom, quantization-aware variants. The resulting hybrid network, referred to as qYOLOv2 in this work, comprises both CNN2SNN-specific and standard Keras layers. Its weights and activations are represented at the selected reduced bit widths, producing a model compatible with subsequent conversion to an Akida model. Then, the converter groups the layers from the quantized model into Akida SNN layers (e.g., a sequence of Conv2D, BatchNormalization and ReLU layers is typically collapsed into a single Akida convolutional layer). The sparsity resulting from quantization and ReLU enables Akida to implement computations using a biologically inspired, event-based paradigm. The converted network, henceforth referred to as sYOLOv2, uses integer-valued inputs and weights and performs integer operations in the Akida runtime. A spike is generated for a neuron when a threshold of activation is reached. To generate an output, all neurons of a Neural Processing Unit (NPU) in the chip must reach the threshold. This feature is key to achieving high efficiency [28]. The converted sYOLOv2 model is executed with a single inference pass (one inference timestep) per input sample; consequently, it does not involve the multi-timestep temporal integration and associated latency–accuracy trade-off commonly found in SNNs [39].

To quantize the model with CNN2SNN toolkit, three bitwidth parameters must be defined: (i) the weight precision for the first layer, (ii) the weight precision for all subsequent layers, and (iii) the activation precision. The separation between the weights of the first layer and the rest reflects the conversion behavior, wherein the first convolution is mapped to an Akida V1 “InputConvolutional” layer—the only Akida V1 layer that supports 8-bit weights and raw image inputs (RGB or grayscale). All remaining Akida V1 convolutional layers accept 1-, 2-, or 4-bit 3D input tensors. In our configuration, frame-based and event-based models used 8-bit precision for the first (input) layer to preserve information from raw data, and 4-bit precision for the remaining weights and activations. For the tensor-based model, all three quantization parameters were set to 4 bits, as this model employed eight-channel input and during conversion its first layer is mapped to a standard Akida V1 convolution (rather than the special InputConvolutional layer). Although the Akida V2 software library introduced spatiotemporal convolutions to capture fine-grained temporal dynamics in event-tensor inputs, this functionality is not supported on the currently available AKD1000 hardware targeted in this study, and no compatible alternative exists for Akida V1, which limits temporal-dynamics encoding.

The complexity of the coastal environment makes it difficult to detect people, in many cases even visually. Considering the altitude and distance to the beach of the UAV during data acquisition, the bounding box of a near “beach user” may have a size of 10×30 pixels approximately at full-resolution, which is a very low number of pixels. This implies extremely limited texture and edge evidence per instance. In this small-object setting, extracted features can undersample the target, making the object intrinsically hard to detect and leading to degraded overall performance. Examples for each class are provided in Figure 4. In addition, due to the limitations of the neuromorphic chip, a complex model with the original high-resolution camera input could not be deployed. Instead, the default 224 × 224 resolution was employed as input size for all networks, as a larger size did not allow deployment of the full SNN in the neuromorphic hardware.

Table 2 illustrates the number of neurons in the YOLOv2 and sYOLOv2 networks. The YOLOv2 network exhibits a higher neuron count, with a value close to 1,708,000 neurons. In comparison, the sYOLOv2 network, corresponding to its spiking version, shows a similar number of neurons, approximately 1,706,000. This small difference indicates minor structural changes due to network adjustment when designing the spiking counterpart.

Regarding the number of layers, in the spiking network, a single layer may perform additional operations that, in the classical network, are typically assigned to separate layers. For example, an SNN convolutional layer may also perform pooling and ReLU operations to the convolution outputs. That is why we can see a major difference in the number of network layers for both computational paradigms (18 for the spiking version, 53 for the classical one). Regarding the network memory storage needed, as shown in Table 2, significant disparity in model size is observed. The baseline YOLOv2 model possesses a network weight of approximately 13.6 megabytes (MB). In contrast, the sYOLOv2 model exhibits a substantially lower network weight of approximately 4.2 MB. This constitutes a 70% size reduction compared to the classical version. The considerably smaller footprint of the sYOLOv2 model is due to the quantization process during network conversion.

### 3.5. Data Preprocessing

As established in the previous section, models designed to accept inputs at the native sensor resolutions (1224 × 1024 and 1280 × 720 for frame and event cameras, respectively) cannot be deployed on the AKD1000. Consequently, a unified preprocessing pipeline was required for all models to ensure a fair comparison across architectures and sensing modalities. Rescaling the frame and event inputs to the chip-compatible 224×224 would suppress fine spatial structure essential for person detection. Instead, we employed a sliding-window approach whereby each full-resolution frame or event slice was partitioned into 56 partially overlapping patches of size 224 × 224, with inference performed independently on each patch. The 56 patches were concatenated into a single batch and submitted to the inference backend in one prediction call. The resulting predictions were then decoded separately for each patch, and the predicted bounding-box coordinates were translated to the corresponding locations in the original full-resolution image using each patch’s spatial offset. Predictions from all patches were subsequently concatenated. Because partially overlapping patches may produce multiple detections for the same person, non-maximum suppression was applied to remove redundant bounding boxes (those with an overlap of more than 70%). The remaining detections, expressed in the coordinate system of the complete image or event slice, were used for the final evaluation. This procedure preserves the effective spatial resolution presented to the model while standardizing inputs across models, at the cost of increased computational load and reduced throughput.

### 3.6. Model Training

YOLOv2 training was conducted on an NVIDIA RTX A6000 GPU under Ubuntu 20.04 LTS running on Windows 10 via WSL2, using TensorFlow 2.15 and Keras 2.15 with CUDA 12.2. The YOLOv2 models for the three input representations were optimized with Adam [40] with a constant learning rate of $1\times 10^{-4}$, while all remaining optimizer hyperparameters followed the Keras defaults. The detection loss followed the formulation in the original YOLO paper [35]. Training was run for a maximum of 100 epochs with early stopping based on validation loss: training continued only when the validation loss decreased by at least $10^{-3}$ relative to the best value observed so far, and it was terminated if no such improvement occurred for 10 consecutive epochs, after which the best-performing weights were retained. Mini-batch sizes were 50 for frame and event inputs and 32 for tensor inputs. Subsequently, the quantized YOLOv2 model (qYOLOv2) was fine-tuned for five additional epochs to recover the accuracy loss introduced by quantization, following the procedure described in the BrainChip CNN2SNN documentation for Akida V1 [38]. This post-quantization training stage is referred to as quantization-aware training (QAT). The spiking variant (sYOLOv2) was not specifically trained, as it could be obtained exclusively via ANN to SNN conversion of the corresponding trained YOLOv2 model.

As can be observed in Table 1, the dataset is moderately imbalanced toward the “beach user class” (in the training set: there was approximately 66% of beach users vs. 17% of bathers and 17% of surfers). To mitigate the potential bias to the majority class, we followed an augmentation strategy during training to effectively increase the variability of all classes. Each sample in a batch first received geometric transformations (horizontal flip, slight translations, and mild affine transformations). These transformations were applied for all data types. Additional augmentations were then sampled independently with a probability of 0.5 per operation, using modality-specific sets. For frames, these augmentations were blurring (Gaussian, average, or median), sharpening, additive Gaussian noise, pixel dropout (up to 10% of pixels set to 0), and brightness and contrast adjustments. For event and tensor data, fewer operations were applied to avoid information loss; event data received pixel dropout only, whereas tensor data received per-channel pixel dropout together with random channel shuffling (i.e., permutation of tensor channels).

### 3.7. End-to-End Full-Resolution Processing Pipeline

The complete processing pipeline comprises input loading, patch extraction, backend inference, reconstruction of full-image detections, and postprocessing through non-maximum suppression (NMS).

Depending on the evaluated input modality, the pipeline loaded either as a grayscale image (for frames and events) or a precomputed temporal tensor (eight-channel numpy array). Frame-based classical inputs had a native resolution of 1224×1024 pixels, whereas the event-based frames and temporal tensors had a resolution of 1280×720 pixels. No resizing was applied before patch extraction, thereby preserving the spatial detail required for small-object detection.

Each full-resolution sample was partitioned into overlapping 224×224 patches using a sliding-window procedure. The patch origins were retained to map the detections back to the original image coordinates. The implementation was configured to generate a fixed grid of 56 patches for every input modality. The 56 patches were concatenated into a single batch that was sent to the inference backend. For the conventional models, the batch was converted to a TensorFlow tensor and executed on either the Jetson Orin Nano CPU or GPU. For sYOLOv2, inference was performed using the Akida runtime on either the CPU simulation or the AKD1000.

The output corresponding to each patch was subsequently decoded and their coordinates were translated to full-image coordinates using the stored patch origin. Finally, NMS removed duplicated detections using an IoU threshold of 0.7. The remaining bounding boxes with a confidence score of at least 0.7 constituted the final set of detections retained for the full-resolution image.

### 3.8. Evaluation Metrics

Rather than reporting model or hardware results in isolation, we quantify the contribution of each subsystem and the interactions among subsystems to evaluate the resulting end-to-end performance. This perspective enables a more faithful assessment of how design choices propagate through the pipeline.

This approach encompasses the following aspects:

1. **Variability in acquisition systems**: Multiple sensing technologies have been considered (frame-based and event-based sensors) to capture how acquisition choices affect downstream metrics.
2. **Diversity in input data types**: Both frame-based inputs (standard images and frame representations from event data sequences) and tensor-based spatio-temporal representations were evaluated, enabling analysis of how input characteristics affect accuracy and efficiency.
3. **Evaluation on multiple computation platforms**: Performance and efficiency are assessed across a variety of hardware platforms to characterize performance and efficiency across the evaluated model–platform configurations.

#### 3.8.1. Task Related Metrics

A set of metrics was selected to assess task performance; these metrics are standard for evaluating the performance of classification and object detection algorithms. In a detection task, true positives (TP), false positives (FP), and false negatives (FN) are defined by matching ground truth and algorithm prediction bounding boxes: a true positive is a prediction that validly matches a single ground-truth bounding box; a false positive is a prediction that does not match any ground-truth bounding box; and a false negative is a ground-truth bounding box with no corresponding prediction. The following metrics were employed:

- **Precision**: The proportion of correct predictions among all positive predictions made by the model. A precision value above 0.5 indicates that a majority of the model detections were correct.
  (1) $$\mathrm{Precision}=\frac{\mathrm{TP}}{\mathrm{TP}+\mathrm{FP}}$$
- **Recall**: The proportion of true positives that were correctly identified by the model. A recall value above 0.5 indicates that the model detected a majority of labeled instances.
  (2) $$\mathrm{Recall}=\frac{\mathrm{TP}}{\mathrm{TP}+\mathrm{FN}}$$
- **F1-Score**: The harmonic mean of precision and recall. This metric summarizes the trade-off between them, and it is particularly informative under class imbalance. A value of 1.0 indicates perfect precision and recall, while 0.0 would be obtained if either the precision or the recall is zero.
  (3) $$F_{1}=2\cdot \frac{\mathrm{Precision}\cdot \mathrm{Recall}}{\mathrm{Precision}+\mathrm{Recall}}$$
- **Intersection Over Union (IoU)**: This metric measures the proportion of overlap between the predicted and ground truth bounding boxes. A value of 1.0 indicates perfect alignment, while 0.0 indicates no overlap. A detection was considered a true positive when the IoU between the predicted and ground truth bounding boxes was 0.5.
  (4) $$\mathrm{IoU}=\frac{\mathrm{Area}\;\mathrm{of}\;\mathrm{Overlap}}{\mathrm{Area}\;\mathrm{of}\;\mathrm{Union}}$$
- **Mean Intersection Over Union (mIoU)**: This is the average IoU computed across all bounding boxes. It was calculated considering only true positives (IoU values of at least 0.5).
  (5) $$\mathrm{mIoU}=\frac{1}{N}\sum_{i=1}^{N}{\mathrm{IoU}}_{i}$$
  The overall mIoU was calculated as the mean of the class-wise mIoU values. Since class-wise mIoU is computed only from true-positive detections (IoU ≥0.5), classes with no true positives have an undefined mIoU and were therefore excluded from the overall average.
- **Average Precision (AP):** This metric summarizes the precision–recall relationship for each class as the area under its interpolated precision–recall curve:
  (6) $$\mathrm{AP}=\int_{0}^{1}p_{\mathrm{interp},c}\left(r\right)\;dr$$
  where $p_{\mathrm{interp},c}\left(r\right)$ is the interpolated precision for class *c* at recall *r*. The curve was constructed from predictions ranked by confidence. The reported metric corresponds to AP_50_, as the IoU threshold for matching was 0.5.
- **Mean Average Precision (mAP):** Represents the average of the AP values obtained for the *C* evaluated classes:
  (7) $$\mathrm{mAP}=\frac{1}{C}\sum_{c=1}^{C}{\mathrm{AP}}_{50}c.$$

For the operating-point metrics, including F1-score, precision, recall, and mIoU, detections were filtered using a confidence threshold of 0.7. This threshold was selected to provide a balance between retaining detections and limiting the number of bounding boxes generated, which is particularly relevant for the latency of the system. Average precision (AP_50_) was instead evaluated over the complete range of confidence scores, retaining predictions down to a minimum confidence threshold of 0.001 to construct the full precision–recall curve.

#### 3.8.2. Efficiency Metrics

Efficiency was evaluated for both acquisition sensors and detection models. The power consumption metrics were measured using the jtop tool from the jetson-stats software package [41], which was developed for controlling and monitoring several NVIDIA Jetson boards. This tool enables reading from the three-channel INA3221 power monitor, with the first channel being (“VDD_IN”), the one employed in this work to measure the System on Module (SoM) input power consumption. The tool was configured to sample energy consumption every 20 ms. For time related metrics, the time module from Python 3.10 was employed. To obtain representative estimates of the efficiency and power consumption, the test set was processed five times. Before each measured run, ten unmeasured prediction rounds were executed to warm-up the corresponding backend and exclude recurrent first-call initialization effects from the processing metrics. The efficiency analysis was divided into preprocessing, backend execution and postprocessing phases. Next, the efficiency of the end-to-end execution—including all three phases—was also analyzed. In particular, the following efficiency metrics were considered:

- **Preprocessing time (ms/full-resolution sample)**: Time required to read the input image or tensor, divide it at its native resolution into 56 patches, and concatenate these patches into the batch submitted to the backend.
- **Backend execution time (ms/full-resolution sample)**: Combined time required for device transfer and model prediction, including result materialization or retrieval.
- **Postprocessing time (ms/full-resolution sample)**: Time required to decode the predictions generated for the 56 patches, transform the predicted bounding boxes into full-image coordinates, and apply non-maximum suppression.
- **Backend throughput (full-resolution samples/s)**: Number of complete 56-patch batches processed by the backend per second, calculated as $\left[{\frac{1}{T}}_{backend}\right]$, where T_*backend*_ is the mean backend execution time per full-resolution sample. This metric excludes preprocessing and postprocessing.
- **Wake-up time (s)**: The average time required by the system until completion of the first warm-up prediction. This measure included the time for software-library and runtime initialization, model loading, loading of the first input, construction of its first 56-patch batch, and execution of the first prediction call. The first prediction was included because importing and loading the software libraries does not necessarily initialize all inference-backend resources. Depending on the selected backend, the first call may trigger TensorFlow graph tracing and concrete-function creation, CUDA context initialization, lazy loading of CUDA modules and kernels, cuDNN sub-library loading, and initial backend memory allocation [42,43,44]. These operations are subsequently cached or reused and therefore are not representative of steady-state inference latency.
- **Average Orin Nano power consumption (mW)**: Mean power consumed by the Jetson Orin Nano platform over the period required to process the full test set, including preprocessing, backend execution, and postprocessing steps. This is a gross SoM measurement and excludes startup and warm-up periods. For AKD1000 experiments, it includes the activity of the Orin Nano host during communication with the accelerator but not the separately measured power consumed internally by the AKD1000 chip.
- **AKD1000 backend power consumption (mW)**: Mean power consumed internally by the AKD1000 during backend execution after each measured prediction. This is a chip-level backend measurement and therefore has a different boundary from the Orin Nano end-to-end power measurement. It excludes Orin Nano host consumption and AKD1000 consumption outside the prediction call.
- **End-to-end energy (mJ/full-resolution sample)**: Mean energy required to process one full-resolution sample across the complete measured pipeline. For CPU and GPU configurations, it corresponds to the integrated Orin Nano “VDD_IN” energy during preprocessing, backend execution, and postprocessing. For the AKD1000 configuration, it is calculated as the sum of the Orin Nano end-to-end energy and the AKD1000 chip energy associated with processing the 56 patches.

For the sensors, a current consumption was measured using a CurrentRanger meter (LowPowerLab, Canton, MI, USA) [45], recording two operating conditions: the current consumed during active data capture and the current consumed in an idle or standby state.

Statistical Analysis: The statistical significance of all measured metrics was evaluated using five independent runs of the complete test set, with each run considered as an independent statistical sample (*n* = 5). Statistical comparisons were performed using a two-way ANOVA. Normality and homogeneity of variances were assessed using the Shapiro–Wilk and Levene tests, respectively. Both tests indicated deviations from the ideal ANOVA assumptions, which is expected given the heterogeneous hardware platforms evaluated. When statistically significant effects were observed, Tukey’s HSD post-hoc tests were applied. Results are reported as mean ± standard deviation together with 95% (*p* < 0.05) confidence intervals and exact *p*-values for all key results. All analyses were implemented in MATLAB 2021b.

#### 3.8.3. Model Related Metrics

Architectural complexity of the networks was summarized by the number of layers, the number of neurons, the number of neurons per layer, and model size, which together describe the interconnection structure and indicate expected computational and memory demands.

In addition, sparsity was analyzed for sYOLOv2. In Spiking Neural Networks (SNNs), sparsity can refer to several aspects, including the spatial distribution and temporal density of input events, as well as the sparsity of internal network activity. In this study, however, the reported value for sYOLOv2 was obtained from the Akida runtime [46] and corresponds to the mean, across network layers, of the proportion of zero-valued output activations. It therefore characterizes internal layer-output sparsity rather than the spatial or temporal sparsity of the input representation.

## 4. Results

### 4.1. Performance Results

The comparative analysis of performance metrics (F1-score, precision, recall, AP_50_, mAP_50_ and mIoU) for the YOLOv2, qYOLOv2 and sYOLOv2 architectures, considering three different input data types (frames, event-based data, and temporal tensors), reveals clear behavioral patterns overall and for each class (Table 3 and Table 4). To provide a contemporary reference for this detection task, an additional experiment was conducted using YOLOv26, the most recent YOLO version available at the time of this study. YOLOv26 [47,48] was trained and evaluated using its conventional processing scheme for this task, using both full-resolution frame-based images and accumulated event-image inputs. In both cases, the single-channel inputs were replicated across the three input channels required by the standard YOLOv26 architecture. The resulting images were resized to 1024 × 1024 and processed as complete images, rather than using the 224 × 224 sliding-window pipeline employed for the YOLOv2-derived models. The eight-channel temporal tensor representation was not included in this reference experiment because preserving its temporal channels would require modifying the standard YOLOv26 input architecture and independently validating a tensor-specific model variant, which would no longer constitute the same standard reference configuration. This experiment was intended to assess how a current state-of-the-art YOLO detector performs when addressing the same detection problem and to contextualize the results obtained with YOLOv2 and sYOLOv2.

Overall, models trained with tensor-based data tend to perform worse than those trained on frames or event-based data. For the classic YOLOv2 model, the highest F1-score was achieved with frame inputs (0.215), while the lowest corresponded with tensor data (0.114). A similar trend was observed in qYOLOv2 and sYOLOv2, where event data achieved the best F1-score (0.168 and 0.172), outperforming both frames (0.155 and 0.154) and tensor inputs (0.080 and 0.085). In terms of mIoU, all models achieve relatively high and consistent values across all data types, ranging from 0.608 to 0.648. sYOLOv2 achieved higher overall mIoU than YOLOv2 for event input (0.647 vs. 0.634). For sYOLOv2, event inputs improved recall relative to frame inputs (0.184 vs. 0.130), while precision decreased from 0.189 to 0.161. In contrast, YOLOv2 exhibited its highest precision (0.227) with frames and highest recall (0.209) with events. The mAP_50_ results followed a comparable pattern: YOLOv2 obtained its highest overall value with frame input (0.212), whereas qYOLOv2 and sYOLOv2 achieved their highest mAP_50_ values with event input (0.111 and 0.114, respectively). Tensor inputs produced substantially lower mAP_50_ values for all models (0.020–0.029). Moreover, qYOLOv2 and its spiking conversion, sYOLOv2, produced closely comparable results across all metrics and input modalities; their overall F1-score, mAP_50_, and mIoU differed only marginally for frames, events, and tensors. The event-based sYOLOv2 configuration exhibited lower F1-score and mAP_50_ than YOLOv2, while preserving a similar mIoU. Example images of inference performed by YOLOv2 classic and sYOLOv2 are shown in Figure 5.

At the class level (Table 4), Bather was consistently the easiest class to detect. YOLOv2 achieved an F1-score of 0.444 and an AP_50_ of 0.546 with frame input. Although qYOLOv2 and sYOLOv2 obtained lower values, Bather remained their strongest class, with their highest AP_50_ values obtained for frame input (0.262 and 0.264, respectively).

Conversely, Surfer was the most difficult class across all three pipelines. For YOLOv2, its best F1-score and AP_50_ were only 0.074 and 0.011, respectively; for qYOLOv2 and sYOLOv2, AP_50_ did not exceed 0.015 and F1-score did not exceed 0.079. Beach User showed intermediate performance across the YOLOv2-derived models and input modalities. Across these configurations, its F1-score ranged from 0.085 to 0.217 and its AP_50_ from 0.019 to 0.101, placing it consistently between the stronger Bather results and the substantially lower Surfer results.

YOLOv26 achieved its highest overall performance with frame input, with an F1-score of 0.323, an mAP_50_ of 0.465, and an mIoU of 0.712. Its precision was particularly high (0.912), although recall remained relatively limited (0.196). With event input, YOLOv26 obtained an F1-score of 0.024, an mAP_50_ of 0.268, and an mIoU of 0.676. As observed with frame input, precision remained high (0.863), whereas recall decreased markedly to 0.012. The low F1 score and recall values obtained should be interpreted in relation to the conservative confidence threshold of 0.7 used for the operating-point metrics. At this threshold, YOLOv26 achieved high precision but limited recall, indicating that the model produced comparatively few false-positive detections while missing a substantial proportion of the targets. This effect was particularly pronounced for event input, where the very low recall strongly reduced the F1-score despite the high precision. Its mAP_50_ result, calculated over the full precision–recall curve, provides a less threshold-dependent assessment. In this metric, YOLOv26 reached 0.465 with frames and 0.268 with events, indicating that the event-based model retained meaningful detection capability across confidence thresholds despite its poor operating-point performance at 0.7. At the class level, Bather was again the easiest class to detect, reaching an AP_50_ of 0.771 with frames and 0.411 with events, followed by Beach User with AP_50_ values of 0.415 and 0.282, respectively. Surfer remained the most challenging class, with AP_50_ values of 0.208 for frames and 0.110 for events. Compared to YOLOv2, YOLOv26 increased overall mAP_50_ from 0.212 to 0.465 for frames and from 0.133 to 0.268 for events, while also providing higher AP_50_ for all three classes in both representations.

#### Quantization-AwareTraining Analysis

To assess the effect of quantization-aware training (QAT) on detection performance, the quantized YOLOv2 model was further trained for five additional epochs using each of the three input representations: frames, events, and tensors. The resulting models were evaluated on the test set and compared with the corresponding original and quantized models. Table 5 reports the overall results for the original YOLOv2 model, its quantized version (qYOLOv2), the corresponding SNN-converted model (sYOLOv2), the quantized model fine-tuned for five additional epochs (qYOLOv2-QAT), and its SNN-converted counterpart (sYOLOv2-QAT). This comparison makes it possible to evaluate the effect of quantization-aware fine-tuning before and after conversion to an SNN across frames, events, and event-tensor inputs. The QAT variants were analyzed separately and were not used for modality-dependent model selection in the main comparison presented in Table 3 and Table 4, for which the non-QAT qYOLOv2 and sYOLOv2 variants were used consistently across all three input representations.

Table 5 shows that the performance loss associated with quantization depends on the input representation and on whether QAT is applied. For frame inputs, the non-QAT qYOLOv2 and sYOLOv2 models provided the best quantized results. Relative to YOLOv2, F1-score decreased from 0.215 to 0.155 and 0.154, respectively, while mAP_50_ decreased from 0.212 to 0.105 and 0.106. Applying QAT to frames further reduced F1-score to 0.135 and mAP_50_ to 0.082–0.080. A similar pattern was obtained for tensor inputs: qYOLOv2 and sYOLOv2 retained F1-scores of 0.080 and 0.085, respectively, compared with 0.114 for YOLOv2, while QAT resulted in F1-scores of 0.077 and 0.078 and did not improve mAP_50_.

For event inputs, QAT produced the strongest quantized models. Without QAT, qYOLOv2 and sYOLOv2 obtained F1-scores of 0.168 and 0.172, respectively, and mAP_50_ values of 0.111 and 0.114, both below the corresponding YOLOv2 values of 0.201 and 0.133. After QAT, performance increased to F1-scores of 0.202 for qYOLOv2-QAT and 0.205 for sYOLOv2-QAT, with mAP_50_ values of 0.153 and 0.151. These results match, or slightly exceed, the original YOLOv2 F1-score and mAP_50_ for event data, while maintaining comparable mIoU values. Across all modalities, conversion of each quantized model to its spiking counterpart caused only minor changes in the evaluated metrics. Thus, the sYOLOv2 and sYOLOv2-QAT models closely preserved the performance of their corresponding qYOLOv2 and qYOLOv2-QAT versions.

### 4.2. Efficiency Results

The efficiency analysis evaluated all model–platform configurations that were feasible within the corresponding execution frameworks. YOLOv2 was retained as a standard Keras model, whereas qYOLOv2 was a Keras model containing CNN2SNN quantized layers together with compatible standard Keras layers; both were therefore executed using the TensorFlow backend on the Jetson Orin Nano CPU (referred to as YOLOv2-CPU and qYOLOv2-CPU, respectively) and GPU (named as YOLOv2-GPU and qYOLOv2-GPU, respectively). In contrast, the converted Akida model, sYOLOv2, was evaluated using either the CPU-based software simulation of the Akida processor model (sYOLOv2-CPU) or the AKD1000 accelerator (sYOLOv2-AKD1000). Before evaluating the model–platform configurations, the power consumption of Jetson Orin Nano platform was measured in idle mode (without performing any processing and computation) with and without connecting the AKD1000 chip through PCIe for a period of 30 min. It was observed that connecting AKD1000 only increased power consumption of Jetson Orin Nano by 283 mW (from 4687 mW to 4970 mW). This increment represented host-side overhead associated with the attached PCIe device and did not represent the power consumed internally by the accelerator. The AKD1000 chip-level idle power, measured separately through the Akida runtime, was 670 mW.

Table 6 separates the contribution of input preprocessing, backend execution, and output postprocessing. For the original YOLOv2 model, frame and event inputs produced similar backend execution times on both CPU (626.973 and 629.663 ms per full-resolution sample, respectively) and GPU (205.667 and 206.349 ms). The observed differences were below 0.5% and were not statistically significant (p>0.05). Consequently, the GPU backend was approximately 3.0 times faster than the CPU backend for these two representations. However, this acceleration was reduced at the end-to-end level (Table 7) because preprocessing and postprocessing cannot benefit equally from GPU execution. With frame input, for example, the YOLOv2–GPU backend processed 4.862 full-resolution samples/s, whereas the complete pipeline achieved 2.011 samples/s; preprocessing and postprocessing together represented approximately 59% of the corresponding pipeline latency. For event input, postprocessing alone accounted for approximately 58% of the end-to-end latency, reducing throughput from 4.846 samples/s at the backend to 1.923 samples/s for the complete pipeline.

Input representation also affected the different stages of the pipeline. Event inputs consistently required the shortest preprocessing times across all model-platform combinations (*p* < 0.001), whereas tensor preparation required between 74.576 and 129.934 ms, depending on the model and platform. Tensors also showed higher mean backend execution times than frames and events. For YOLOv2, tensor backend execution was approximately 16% slower on CPU and 42% slower on GPU than frame execution. The effect was particularly pronounced on the AKD1000, where tensor processing required 2443.614 ms, compared with 1549.070 ms for frames, an increase of approximately 58%. These three increases were statistically significant (p<0.001). Therefore, the temporal information contained in the eight-channel tensors did not translate into greater computational efficiency under the evaluated implementation.

The quantized ANN did not provide an execution-time or energy advantage on the Jetson CPU or GPU. Compared with YOLOv2, qYOLOv2 increased backend execution time by approximately 50–59% on CPU and 27–57% on GPU, depending on the input representation. Its end-to-end energy was also higher for every corresponding configuration. All increases in backend execution time and end-to-end energy were statistically significant (p<0.01). In particular, qYOLOv2–GPU required 5176.707 mJ per frame sample, compared with 2886.551 mJ for YOLOv2–GPU.

The end-to-end results in Table 7 identify YOLOv2–GPU as the configuration with the lowest energy requirement for all three input representations. It achieved 2.011, 1.923, and 1.575 full-resolution samples/s for frames, events, and tensors, respectively, with corresponding energy requirements of 2886.551, 3117.238, and 3982.227 mJ per sample. Its frame and event throughputs were the highest among the evaluated configurations. For tensor inputs, qYOLOv2–GPU achieved a marginally higher mean end-to-end throughput (1.578 samples/s), but this difference was not statistically significant (p=0.782), and its energy requirement was approximately 50% greater. Although the YOLOv2–GPU backend was approximately 2.5–3.1 times faster than its CPU counterpart, its end-to-end throughput advantage was reduced to approximately 1.4–1.5 times because host-side preprocessing and postprocessing represented a substantial fraction of the complete pipeline latency.

For the spiking model, AKD1000 execution did not outperform CPU execution in terms of backend throughput. For frame, event, and tensor inputs, sYOLOv2–AKD1000 achieved 0.646, 0.608, and 0.409 backend samples/s, respectively, compared with 0.685, 0.704, and 0.622 samples/s for sYOLOv2–CPU. The AKD1000 configuration also required more end-to-end energy than the CPU implementation: 14596.105 versus 9016.226 mJ for frames, 15019.201 versus 9512.322 mJ for events, and 20845.101 versus 10863.365 mJ for tensors. The isolated AKD1000 backend consumed approximately 1.22–1.36 W, but these values must not be directly compared with the Orin Nano end-to-end power column because they correspond to different devices and measurement windows. All differences in backend throughput and end-to-end energy were statistically significant (p<0.01). The combined end-to-end energy, which includes both the host and the AKD1000 contribution, is therefore the appropriate metric for comparing the complete configurations.

The CPU configurations provide the most direct controlled comparison because the same processing platform was used for the three models. For frames, events, and tensors, YOLOv2 consumed 6.158, 6.049, and 6.118 W, respectively, with corresponding end-to-end energy costs of 4.707, 4.678, and 5.562 J per sample. qYOLOv2 increased average power to 9.265, 9.033, and 9.014 W and energy consumption to 10.311, 10.557, and 11.245 J. Relative to YOLOv2, this represents increases of 47.3–50.5% in power and 102.2–125.7% in energy. sYOLOv2 consumed 5.730, 6.040, and 6.166 W and required 9.016, 9.512, and 10.863 J per sample. Compared with qYOLOv2, sYOLOv2 reduced average power by 31.6–38.2% (p<0.05). Its mean energy consumption was also 3.4–12.6% lower, although these differences were not statistically significant. Compared directly with YOLOv2, sYOLOv2 exhibited similar average power, ranging from a 7.0% reduction to a 0.8% increase, none of which were statistically significant. However, its energy consumption was 91.5–103.4% higher (p<0.05), consistent with its approximately 48.4–51.4% lower end-to-end throughput.

Wake-up time exhibited large differences between software stacks and model formats. The shortest values were obtained for sYOLOv2–CPU and sYOLOv2–AKD1000 with frame and event inputs, ranging from 7.622 to 12.838 s, whereas YOLOv2 required 48.394–83.768 s and qYOLOv2 required 62.841–93.850 s, depending on the platform and representation. Tensor inputs also increased wake-up time for both sYOLOv2 configurations (all p<0.01), reaching 24.380 s on CPU and 25.103 s on AKD1000.

Overall, among the evaluated configurations, YOLOv2–GPU provided the lowest end-to-end energy for every input representation and the highest throughput for frames and events. Although the AKD1000 exhibited low chip-level backend power, its longer execution time and continued dependence on the Orin Nano host resulted in the highest end-to-end energy requirements among the evaluated spiking configurations. The distributions of the timing and efficiency measurements across the five experimental runs are additionally provided in Figures S1 and S2, respectively.

Regarding the acquisition system, current consumption measurements and estimated power consumption have been conducted for both imaging technologies—frame-based and event-based cameras—during multiple operational states, including standby mode and active capture configuration.

The data in Table 8 reveals a substantial disparity in energy consumption between conventional frame-based image capture technology and neuromorphic camera system (DVS). It was proven that DVS camera consumed considerably less power, between 5.6× and 5.7× less power, than the frame camera.

The sparsity of the spiking version of the network was analyzed (Table 9). The highest sparsity value is observed when using the frame data type (0.61), followed by the event (0.60) and closely by tensor (0.596) data types. It is important to note that these sparsity values represent activation sparsity, calculated as the mean fraction of zero-valued outputs across the applicable Akida layers, rather than the fraction of zero-valued pixels in the input representation [46]. Although the accumulated-event representations are spatially sparse at the input, they are processed as rasterized images by the Akida V1 InputConvolutional layer [49]; conventional convolutions can propagate individual non-zero inputs across several output positions and thereby reduce sparsity in the resulting activation maps. Consequently, event inputs do not necessarily produce greater internal activation sparsity.

## 5. Discussion

This case study presents a systematic comparison of a YOLOv2-based detection network deployed on the CPU and GPU of a Jetson Orin Nano edge device and on a BrainChip AKD1000 neuromorphic accelerator, while also evaluating frame-based and event-based acquisition sensors. The results offer insights regarding architecture choice, data modality, hardware constraints, and overall system efficiency. Among prior benchmarking works, the most task related is the Event-Camera Object Detection presented in NeuroBench [7], which evaluated event-camera object detection on the Prophesee 1MP Automotive dataset [16]. In this benchmark, two algorithms were compared: a Recurrent Event-camera Detector (RED) ANN and a hybrid ANN-SNN, similar to the original RED ANN with Convolutional Long Short-Term Memory (ConvLSTM) layers replaced by Leaky Integrate-and-Fire (LIF) spiking layers. Although that work reported model-level metrics, such as detection precision, memory footprint and processing rate, it did not evaluate differences across computing platforms or sensor acquisition settings. NeuroBench also introduced two system-track benchmarks aimed at evaluating at software and hardware level: Acoustic Scene Classification and Quadratic Unconstrained Binary Optimization (QUBO). The first could be the most related to our study, in which audio clips from the DCASE dataset [50] were classified using a conventional CNN, deployed on an Arduino Nano 33 BLE CPU, and a feed-forward SNN [51], deployed on the Synsense Xylo [52]. For each algorithm–hardware pair authors reported accuracy, execution time, and energy efficiency. However, despite using multiple recording devices in the dataset, that benchmark does not analyze acquisition at the sensor level (although none of the devices are indicated to be neuromorphic).

Regarding the neural network architecture employed, adapting a relatively complex network such as YOLOv2 to spiking hardware (sYOLOv2) proved feasible. However, the implementation of a more complex YOLOv4, although delivering superior accuracy on conventional platforms, exceeded the capacity of neuromorphic hardware used. This highlights the importance of balancing architectural sophistication with resource budgets in embedded, event-driven systems. This need for balance has also been addressed by other authors, who have developed hardware-centric tools to optimize SNN models for their deployment [23,53]. Relatedly, it must be noted that YOLOv2 is an outdated architecture that has been surpassed by more recent YOLO versions, with YOLOv26 being the newest, and by mobile-oriented architectures. YOLOv2 was selected for this case study because it is currently the most compatible detection model for conversion and deployment on the AKD1000 neuromorphic chip. Accordingly, the conclusions drawn in this work should not be interpreted as representative of all classical CNN-based detectors, but rather as a comparison under strict hardware-compatibility constraints imposed by the evaluated neuromorphic platform. To contextualize the efficiency of more recent lightweight detectors, Shakya et al. [54] evaluated several compact YOLO architectures on the Jetson Orin Nano and reported VDD_IN power consumption ranging from 7.6 to 10.4 W, depending on the model and execution configuration, together with inference speedups of approximately 3–5× when using TensorRT instead of PyTorch. Although differences in datasets, input resolutions, and measurement boundaries preclude a direct energy-per-inference comparison with the present results, these values show that modern lightweight architectures do not necessarily achieve efficiency through substantially lower instantaneous power, but through shorter execution times and optimized inference runtimes. Therefore, both the YOLOv2 architecture and the unoptimized classical inference pipeline used in this work—which did not incorporate deployment-oriented optimizations such as ONNX export, TensorRT compilation, or INT8 execution—should be regarded as a hardware-compatibility baseline rather than as a state-of-the-art efficiency solution for conventional edge devices.

For context, a recent benchmark for deep learning for object detection in edge devices—Jetson Orin Nano among them—stated that architectures such as SSD-MobileNet V1 are more energy-efficient and faster in inference than YOLOv8 [55]. In fact, this study reported power consumptions of 0.09 mW/h (324 mJ) for EfficientDet Lite0, 0.01 mWh (36 mJ) for SSD MobileNet V1 and 0.13 mWh (468 mJ) for YOLOv8 nano on Jetson Orin Nano, all lower than the consumptions presented in our work, with SSD-MobileNetV1 markedly lower. This indicates that YOLOv2 is unlikely to be optimal for standard (non-neuromorphic) edge devices and should be viewed here as a hardware-compatibility baseline rather than a state-of-the-art efficiency model.

Detecting people in coastal aerial imagery remains challenging because targets are often small, distant, partially occluded, and embedded in visually heterogeneous backgrounds containing water, waves, sand, and shoreline structures. The YOLOv26 control results show that the use of a contemporary detector improved performance over YOLOv2 models on the present task, with an overall ${mAP}_{50}$ of 0.465 for frame input and 0.268 for event input, compared with 0.212 and 0.133, respectively, for YOLOv2. The larger improvement observed for frame input indicates that replacing YOLOv2 with a more recent detector did not eliminate the observed performance difference between frame and event inputs for the data and detection task considered in this study. Similar differences between frame- and event-based detection have been reported in previous studies, with event-only representations yielding lower detection accuracy than conventional frames under some acquisition conditions and detector configurations [56,57]. However, both modalities provide complementary information, motivating recent frame–event fusion approaches that combine the richer appearance information of conventional images with the high temporal resolution of event data [56,57,58]. Such approaches have demonstrated improved detection performance or robustness over the individual modalities in challenging scenarios, suggesting frame–event fusion as a promising direction for extending the present work. The class-specific results also remained markedly heterogeneous. For frame input, ${AP}_{50}$ reached 0.771 for Bather, but only 0.415 for Beach User and 0.208 for Surfer. For event input, the corresponding values were 0.411, 0.282, and 0.110. Although the frame-based mAP_50_ value is modest in absolute terms, it is consistent with the difficulty reported in benchmarks for long-range and aerial small-object detection. In the TinyPerson benchmark [59], which contains maritime and beach scenes with persons at very small image scales, a Faster RCNN-FPN detector achieved an AP_50_ of 0.4729, increasing to 0.5247 after scale-matched training. Similarly, the UAVDT benchmark [60] showed that R-FCN achieved an AP of 0.3435 under an IoU threshold of 0.7 in unconstrained UAV imagery, substantially below its performance on conventional road-scene benchmarks. These values are not directly comparable because of differences in class definitions, object-size distributions, and evaluation protocols; nevertheless, they contextualize the substantial challenge posed by small, distant targets in aerial imagery.

The overall low performance observed across all models can be attributed not only to the limitations of the YOLOv2 network and the difficulty of the task, but also to the detection approach followed, which treated each frame (or event input data) independently. Several recent models [61,62] have demonstrated that incorporating temporal context, via mechanisms like ConvLSTMs, can significantly improve both accuracy and robustness. Further implementations should consider integrating temporal tracking mechanisms to exploit sequential coherence, thereby improving detection stability and reducing false negatives. Accordingly, the system presented in this work should be regarded as an application-driven case study of the practical constraints associated with the evaluated sensing, model-conversion, and AKD1000 deployment workflow, rather than as a deployment-ready coastal safety solution.

The modality of input data played a significant role in performance. Overall, tensor-based inputs yielded the lowest results for all metrics across models. This is likely due to the limited temporal granularity of the tensor-based representation in visual tasks, which may fail to capture essential spatiotemporal dependencies needed for effective spike-based processing. Additionally, it is possible that the temporal structure of the visual data was not adequately encoded or presented to the network, which limits its ability to exploit temporal dynamics critical to SNN performance. Some authors have addressed this by encoding temporal information within the SNN architecture, allowing the model itself to learn frame-based representations directly from the relevant event streams input [63]. However, such architectures are not compatible with the neuromorphic hardware employed in this work. Future research should explore specific preprocessing methods for event data to mitigate event “flickering” in challenging regions such as water, in order to increase the precision of the system with event data. In this regard, recent research has identified DVS noise as a major challenge, and some have presented algorithms for noise removal from event cameras [64].

In contrast, frame data performed best on the classical network (F1 = 0.215; mAP_50_ = 0.212) followed closely by event-based data (F1 = 0.201; mAP50 = 0.133), while event-based inputs were beneficial for the spiking model (F1 = 0.172 and an mAP_50_ = 0.114) compared with 0.154 and 0.106, respectively, for the frame-based sYOLOv2 configuration. While frame inputs yielded higher precision for YOLOv2 (0.227 versus 0.194 with events), event-based sYOLOv2 obtained higher recall than its frame-based counterpart (0.184 versus 0.130), although precision decreased from 0.189 to 0.161. Nevertheless, the event-based configuration achieved moderately higher F1-score (0.172 versus 0.154) and mAP_50_ (0.114 versus 0.106). Both YOLOv2 and sYOLOv2 achieved comparable mIoU values (0.60–0.65) across all modalities, indicating that spatial localization capabilities remain largely architecture-agnostic. These findings align with the recent literature [65] demonstrating that SNNs, when paired with event-based sensors, can even outperform CNNs under certain conditions while maintaining significantly better energy efficiency. Overall, the CNN consistently outperformed the SNN across most metrics, likely due to its greater architectural maturity and optimization.

Within the evaluated five-epoch fine-tuning procedure, QAT was beneficial only for event inputs: qYOLOv2-QAT and sYOLOv2-QAT maintained or slightly improved the event-based YOLOv2 F1-score (0.202 and 0.205 versus 0.201) and increased mAP_50_ (0.153 and 0.151 versus 0.133). For frame and tensor inputs, QAT reduced performance. The largest performance loss occurred during conversion from YOLOv2 to the quantized model; subsequent conversion from qYOLOv2 to sYOLOv2 produced only minor changes across the evaluated metrics and input representations. This is consistent with the observation by Castagnetti et al. [66] that the accuracy gap between ANNs and SNNs is mainly associated with quantization error. Although sYOLOv2 is only slightly more compact than its classical counterpart (1,706,000 versus 1,708,000 neurons), the spiking models required approximately four times less memory. This difference was expected because of the quantization process applied during conversion to SNNs and represents an advantage for deployment on memory-constrained edge devices. Quantization is also a key step for ANN deployment at the edge [67]; nevertheless, recent approaches such as SpQuant-SNN [68] and NeuronQuant [69] seek to reduce the associated accuracy loss further. However, qYOLOv2 was quantized using the same CNN2SNN software workflow employed for the SNN conversion. This choice was necessary to ensure that the quantized model complied with the constraints required for successful conversion and deployment on the AKD1000 accelerator.

In addition, model performance varied significantly across data setups (Frames, Events, Tensors) and object classes (Beach User, Bather, Surfer). Limited sample diversity, particularly for the Surfer class, negatively affected model performance. As noted in the Dataset section, surfers were observed only at a single beach (El Socorro) and not in every recording from that site, which further constrained intra-class variability and scene diversity. These results highlight the importance of dataset balance and class variability in future evaluations.

The neuromorphic model, sYOLOv2, exhibited substantial internal sparsity across all data modalities (≈0.6). In SNNs, such sparsity is meaningful because computation and memory access scale with spike activity; fewer active elements generally translate into fewer synaptic operations and lower on-chip work. Several works have reported that increasing sparsity is key to improving energy efficiency [70,71]. The sparsity values were similar across representations, ranging from 0.596 for tensor inputs to 0.609 for frame inputs; thus, the corresponding active fraction changed only from 39.1% to 40.4%. This small variation did not produce a monotonic relationship with the measured AKD1000 backend power. Frame and event inputs consumed 1348.4 and 1357.2 mW, respectively, whereas tensor inputs consumed the lowest average chip power (1223.6 mW) despite exhibiting the lowest sparsity. Therefore, the global activation sparsity metric alone does not predict AKD1000 power consumption, which can also depend on the temporal distribution of activity, routing and memory-access patterns, and other implementation-dependent overheads. Moreover, tensor inputs comprise eight channels, whereas frame and event inputs comprise one channel. This higher input dimensionality provides a plausible explanation for their substantially longer backend execution time (2444 ms, compared with 1549 ms for frames), even though their average chip power was lower. Consequently, the corresponding power–time estimates were approximately 2.09, 2.23, and 2.99 J per full-resolution sample for frame, event, and tensor inputs, respectively. These results indicate that, for the evaluated workload, backend duration—partly associated with the higher-dimensional tensor representation—rather than the small inter-representation differences in activation sparsity was the primary determinant of AKD1000 chip energy.

The efficiency analysis provided a detailed characterization of the computational bottlenecks in the evaluated pipelines. Backend execution represented the largest latency contribution, particularly for the CPU and AKD1000 configurations. Preprocessing remained comparatively short for frames and events, whereas tensor inputs introduced a larger preparation cost because of their multi-channel representation. Postprocessing also constituted a relevant contribution, especially for the GPU configurations, where the shorter backend execution time exposed the cost of decoding predictions from 56 patches, reconstructing full-image coordinates, and applying non-maximum suppression. For example, YOLOv2–GPU achieved a backend throughput of 4.862 full-resolution samples/s with frame input, but its end-to-end throughput decreased to 2.011 samples/s after preprocessing and postprocessing were included. This difference demonstrates that backend throughput alone does not fully characterize the operational performance of an embedded detection pipeline, as end-to-end object-detection deployments must also account for host-side preprocessing, postprocessing, and communication overheads [72].

Among the evaluated model–platform configurations, YOLOv2–GPU provided the highest backend throughput for all three input representations, the highest end-to-end throughput for frames and events, and the lowest end-to-end energy consumption in every case. It processed 3.413–4.862 full-resolution samples/s at the backend and 1.575–2.011 samples/s end-to-end, requiring 2.887–3.982 J per full-resolution sample. qYOLOv2–GPU achieved a slightly higher mean end-to-end throughput for tensor inputs (1.578 versus 1.575 samples/s), although this difference was not statistically significant, while requiring approximately 50% more energy. YOLOv2–CPU achieved lower throughput and required 4.678–5.562 J per sample. At the opposite end of the throughput comparison, sYOLOv2–AKD1000 was the slowest configuration for all input representations, achieving 0.409–0.646 samples/s at the backend and 0.345–0.514 samples/s end-to-end. Software execution of sYOLOv2 on the CPU was faster, reaching 0.622–0.704 samples/s at the backend and 0.568–0.636 samples/s end-to-end. Although the YOLOv2–GPU backend was approximately 2.5–3.1 times faster than its CPU counterpart, its end-to-end throughput advantage was reduced to approximately 1.4–1.5 times because preprocessing and postprocessing together accounted for approximately 54–60% of the total pipeline latency. These comparisons characterize complete model–platform configurations and should not be attributed exclusively to their underlying processing devices.

qYOLOv2 provides an intermediate reference for examining the effect of CNN2SNN quantization separately from conversion and execution on the AKD1000. The quantized Keras model did not improve efficiency when executed directly through the TensorFlow CPU or GPU backends. Compared with YOLOv2, qYOLOv2 increased backend execution time by approximately 50–59% on the CPU and 27–57% on the GPU. Its end-to-end energy consumption was also approximately 102–126% higher on the CPU and 50–105% higher on the GPU, depending on the input representation. These findings should not be interpreted as evidence that reduced-precision arithmetic is intrinsically less efficient. The quantize method from the CNN2SNN toolkit replaces standard Keras layers with custom CNN2SNN quantization-aware layers and produces an intermediate model intended for subsequent conversion to the Akida runtime. Direct TensorFlow execution of this model does not guarantee that its nominal low-bit representation is mapped to native INT4 kernels or to an optimized integer-only inference path. Previous work distinguishes simulated quantization from real integer-only deployment and shows that practical latency and energy improvements depend on the correspondence among the quantization scheme, inference runtime, available kernels, and target hardware [73,74,75]. Accordingly, the lower throughput and higher energy consumption observed for qYOLOv2 are consistent with quantization-related overhead or limited exploitation of low-precision hardware, although the specific contribution of these mechanisms was not separately profiled. The qYOLOv2 results must therefore be interpreted as specific to the evaluated CNN2SNN Keras implementation and TensorFlow backend, rather than as a general limitation of quantization.

Conversion to sYOLOv2 and execution on the AKD1000 did not provide an end-to-end efficiency advantage over YOLOv2–GPU. The AKD1000 backend processed 0.409–0.646 full-resolution samples/s, making YOLOv2–GPU approximately 7.5–8.3 times faster at this stage. After host-side preprocessing and postprocessing were included, sYOLOv2–AKD1000 achieved 0.345–0.514 samples/s and required 14.596–20.845 J per sample, approximately 4.8–5.2 times the energy required by YOLOv2–GPU. The internal AKD1000 backend power remained comparatively stable at approximately 1.224–1.357 W. However, low chip-level power did not translate into low system-level energy because the accelerator required considerably longer backend execution and depended on the Orin Nano host for input preparation, communication, result retrieval, and postprocessing. The Orin Nano remained active throughout these operations, with gross end-to-end SoM power between approximately 5.955 and 6.436 W. Thus, the low instantaneous power of the neuromorphic accelerator was insufficient to compensate for its backend latency and the accompanying host activity in the evaluated dense sliding-window pipeline.

The choice of input representation affected the pipeline stages in different ways. Event inputs consistently showed the shortest preprocessing times, whereas tensor representations required the longest preprocessing, reflecting the additional operations needed to load, organize, and divide their multi-channel data into patches. Nevertheless, this reduction was small relative to backend and postprocessing times and did not produce a consistent end-to-end energy advantage. For YOLOv2, frame and event inputs exhibited similar backend performance on both CPU and GPU. Tensor inputs exhibited longer preprocessing times and lower backend throughput than frame and event inputs across all evaluated model–platform configurations. This effect was most pronounced on the AKD1000, where tensor input reduced backend throughput from 0.646 samples/s for frames to 0.409 samples/s and increased end-to-end energy from 14.596 to 20.845 J. These results indicate that the efficiency of event-derived representations depends not only on their sparsity but also on their dimensionality, memory-transfer requirements, and compatibility with the operations implemented by the target backend.

Regarding wake-up time, the qYOLOv2 configurations exhibited the longest application-readiness times, ranging from 62.841 to 65.854 s on the CPU and from 89.956 to 93.850 s on the GPU, consistent with additional initialization associated with the custom quantized Keras model. YOLOv2–CPU and YOLOv2–GPU produced lower mean values, ranging from 48.394 to 60.550 s and from 70.126 to 83.768 s, respectively. In contrast, sYOLOv2–CPU and sYOLOv2–AKD1000 generally achieved shorter application-readiness times, although tensor input increased them to 24.380 and 25.103 s, respectively. This faster readiness may benefit on-demand or event-triggered applications requiring a rapid response.

Previous studies have reported substantial power reductions for AKD1000 relative to embedded GPU platforms [29,30]. However, such results commonly refer to chip-level inference under different models, workloads, input representations, and measurement boundaries. In the present experiments, AKD1000 power was reported separately at chip level, while the Orin Nano host was measured through the VDD_IN channel and the corresponding energy contributions were combined for end-to-end energy accounting. The results demonstrate that favorable accelerator-level power does not necessarily imply favorable system-level energy. In this application, the processing of 56 dense patches, host–accelerator communication, backend latency, and host-side postprocessing prevented the AKD1000 from achieving the efficiency advantages reported for more accelerator-centered or naturally sparse workloads.

Overall, the results lead to three practical conclusions. First, embedded-AI evaluations should report both stage-level timing and end-to-end energy using clearly defined and consistent measurement boundaries. Second, quantization only provides computational benefits when the resulting operations are supported by an optimized low-precision execution path; nominal bitwidth alone is not an adequate predictor of latency or energy. Third, the effectiveness of neuromorphic acceleration depends on the compatibility of the complete workload with the accelerator. Under the dense 56-patch sliding-window pipeline evaluated here, neither the intermediate CNN2SNN quantization nor conversion and execution on the AKD1000 provided an end-to-end energy advantage over floating-point YOLOv2 on the Jetson GPU. Moreover, the classical pipeline was evaluated without deployment-specific optimizations such as ONNX export, TensorRT engine generation, or INT8 inference. These optimizations could further improve the latency, throughput, and energy efficiency of the classical implementation; therefore, the present comparison does not represent its maximum achievable efficiency.

At sensor level, conversely, the event-based acquisition system was shown to be much more efficient, consuming approximately 82% less power than the frame-based system. This significant difference demonstrates the sensor-level power advantage of the event-based acquisition system in the evaluated setup relative to the frame-based camera.

## 6. Conclusions

This work presents an application-driven case study of the BrainChip AKD1000 neuromorphic accelerator integrated with a Jetson Orin Nano for a UAV-based detection application using frame- and event-based sensors in coastal environments. The study examined the original YOLOv2 model, its quantized version, and its conversion to a spiking neural network, using frame, event, and tensor representations on the CPU and GPU of a Jetson Orin Nano and on a BrainChip AKD1000 accelerator. Accordingly, the findings should be interpreted as results for the evaluated model-conversion workflow and complete model–platform configurations. Because direct comparisons between configurations such as YOLOv2-GPU and sYOLOv2-AKD1000 simultaneously involve differences in model representation, numerical precision, execution runtime, and hardware platform, the observed differences cannot be attributed exclusively to the underlying computing paradigm. Key conclusions are as follows:

- **Coastal detection task difficulty:** Person detection in aerial coastal environments remained challenging because targets were often small and distant, in visually heterogeneous scenes. The YOLOv26 control improved the overall mAP_50_ from 0.212 for YOLOv2 to 0.465 for frame input and from 0.133 to 0.268 for event input compared with YOLOv2, showing that a more contemporary detector can improve performance on the present task. However, this modest result confirms that the task remains difficult.
- **Drawback of the full system processing pipeline**: AKD1000 did not provide an end-to-end energy advantage in the evaluated dense sliding-window pipeline. Its average chip-level backend power was comparatively low (1.224–1.357 W), but this measurement covers the AKD1000 during backend execution only. Once the energy required by the Jetson Orin Nano host was included, the sYOLOv2–AKD1000 configuration required 14.596–20.845 J per full-resolution sample and achieved 0.345–0.514 end-to-end samples/s. The longer backend execution time, processing of all 56 patches regardless of event activity, host–accelerator communication, and host-side preprocessing and postprocessing prevented the lower chip-level power from translating into lower system-level energy. This result does not rule out benefits from neuromorphic hardware in other workloads; rather, it shows that they were not realized under the evaluated conditions. In addition, several mechanisms that could potentially improve the efficiency and representational capability of event-based processing were not considered in this study, including activity-gated processing, event polarity, and more sophisticated temporal coding strategies.
- **SNN conversion and deployment**: Although YOLOv2 is an older, now-obsolete architecture, it provided a technically feasible baseline for quantization and conversion to sYOLOv2 within the constraints of the CNN2SNN workflow and the AKD1000 accelerator. The conversion from qYOLOv2 to sYOLOv2 largely preserved detection performance across the evaluated input representations, with only minor changes in F1-score, mAP_50_, and mIoU. However, for the evaluated pipeline, deployment of the resulting spiking model on the AKD1000 did not reduce end-to-end energy consumption relative to the corresponding qYOLOv2 model executed on either the CPU or GPU of the Jetson Orin Nano.
- **Data modality effects**: Frame and event inputs produced closer detection results than the eight-channel tensor representation. For the original YOLOv2 model, frames achieved an F1-score of 0.215 and an mAP50 of 0.212, whereas events achieved 0.201 and 0.133, respectively; tensor inputs were substantially poorer (F1-score of 0.114 and mAP50 of 0.029). This indicates that the tensor representation failed to encode properly visual information of the event data.
- **Impact of quantization:** Within the five additional quantization-aware training epochs considered in this study, QAT was beneficial only for event inputs. qYOLOv2-QAT and sYOLOv2-QAT achieved event-based F1-scores of 0.202 and 0.205, respectively, with mAP_50_ values of 0.153 and 0.151. In contrast, QAT did not recover the frame- or tensor-based performance loss. However, these results do not exclude the possibility that extending the QAT procedure could improve performance for these input representations, as observed for event inputs. Overall, the largest loss occurred during quantization.
- **Acquisition system efficiency**: The event-based camera demonstrated significantly lower power consumption, approximately a 5.6–5.7× reduction, compared with the conventional frame-based sensor. This energy advantage, combined with the asynchronous and sparse nature of event data, reinforces the suitability of neuromorphic sensing for low-power edge applications.
- **Resource efficiency**: Resource efficiency remains an important factor in embedded deployments. The sYOLOv2 architecture, with a nearly threefold reduction in size compared to YOLOv2, is better suited for devices with strict memory constraints and offers the fastest wake-up times, making it attractive for event-driven or low-duty-cycle operation. However, for continuous, high-throughput workloads, our measurements show that the YOLOv2 GPU pipeline remains the most energy-efficient option, achieving the highest sustained throughput and the lowest energy per full-resolution inference within the complete processing pipeline.

In future work, we will pursue further optimizations of spiking neural networks to facilitate their seamless deployment on neuromorphic hardware and to determine whether the efficiency gains promised by these systems can be realized in practice. Planned refinements include architectural adjustments within hardware constraints—such as permissible layer depth and per-layer channel/neuronal widths. In addition, the observed variability in AKD1000’s power and energy consumption—especially with tensor inputs—highlights the need for better preprocessing optimization and perhaps dedicated hardware pathways. Future work should explore improving data-transfer efficiency, integrating preprocessing accelerators, exploiting pipelined execution strategies, and further evaluating event-based pipelines in combination with neuromorphic inference engines. Another potential avenue for enhancing the performance of this technology lies in preprocessing and optimizing the use of event-based data through proper structuring along the temporal dimension. Future work will investigate event tensor-encoding strategies that remain compatible with hardware constraints while better exploiting temporal dynamics, including a systematic analysis of the optimal number of temporal steps for event-tensor data. For event-only applications, the preprocessing pipeline will be redesigned to enhance event-specific properties—for example, suppressing or skipping regions with insufficient event activity to avoid unnecessary computation. In addition, frame–event fusion represents a relevant direction for exploiting the complementary characteristics of both sensing modalities. Future work should investigate whether combining conventional frames and event data can improve detection robustness under challenging coastal conditions. Overall, the results indicate that substantial opportunities remain to refine the event-based processing and neuromorphic deployment pipeline and to further evaluate its performance and efficiency under different design choices.

## Figures and Tables

**Figure 1:**
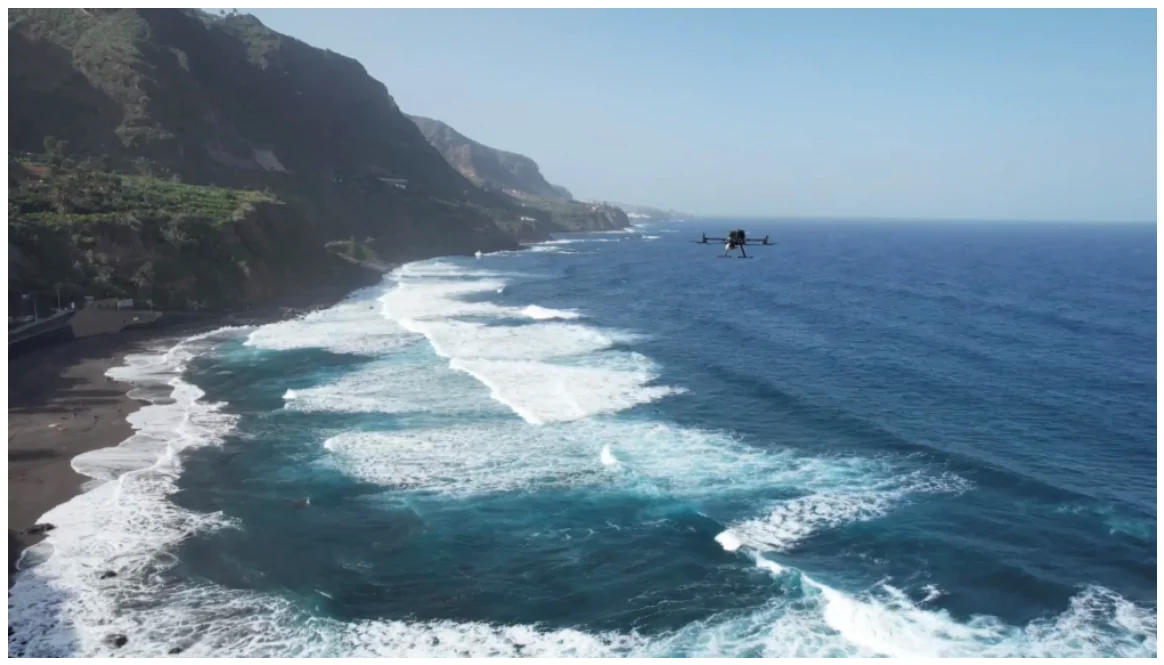
DJI Matrice 350 RTK UAV equipped with synchronized frame-based and event-based cameras for aerial data acquisition flying over El Socorro beach.

**Figure 2:**
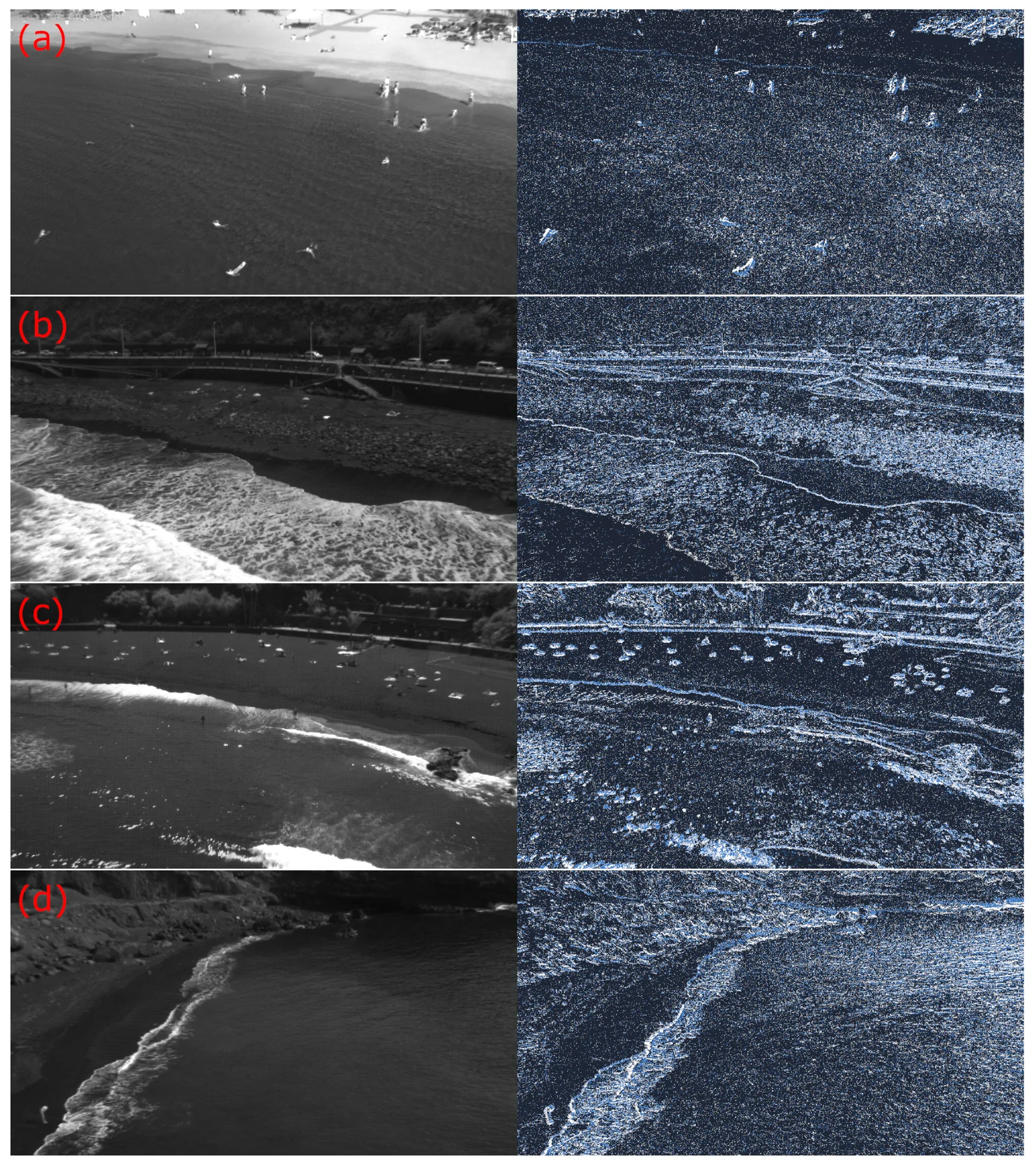
Coastal dataset overview illustrating the diversity of acquisition sites, including frame-based images (**left**) and corresponding event data (**right**) for the four beaches considered: (**a**) Teresitas, (**b**) Socorro, (**c**) Jardín, and (**d**) El Abrigo.

**Figure 3:**
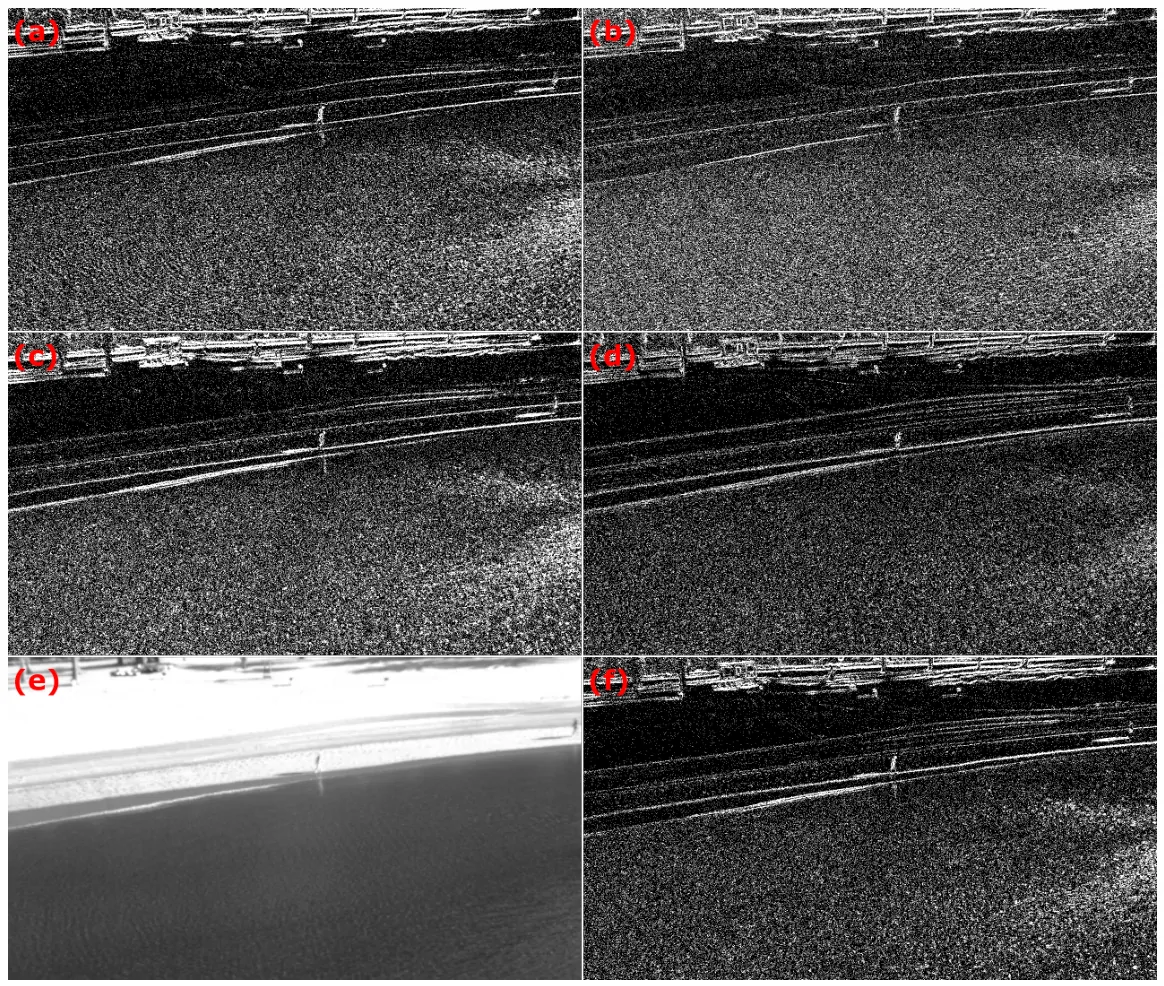
Time-based tensor representation of event data, where asynchronous events are discretized into eight temporal steps encoded as separate input channels. The top two rows (**a**–**d**) show steps 1, 4, 6, and 8 of the process. The bottom row displays the corresponding (**e**) frame-based and (**f**) event-based images.

**Figure 4:**
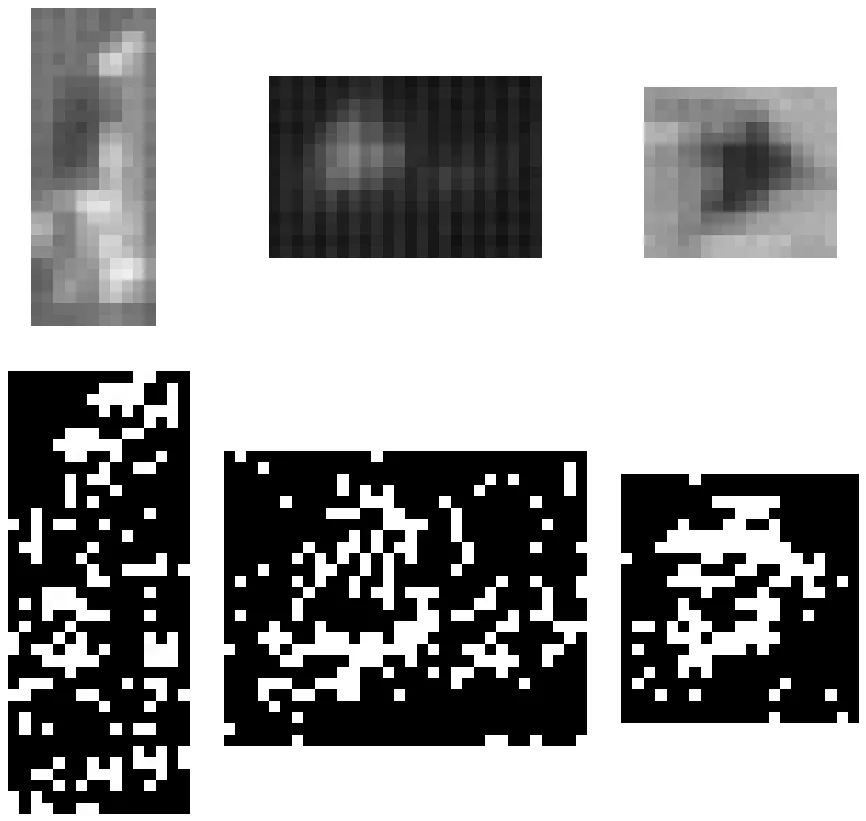
Example of objects on frames (**top**) and event data (**bottom**) for each class: beach user (**left**), bather (**center**) and surfer (**right**). Object dimensions, ordered from left to right and top to bottom, are 11×28, 24×16, 17×15, 16×39, 32×26, and 21×22 pixels. These dimensions highlight the small-object detection challenge in coastal UAV imagery.

**Figure 5:**
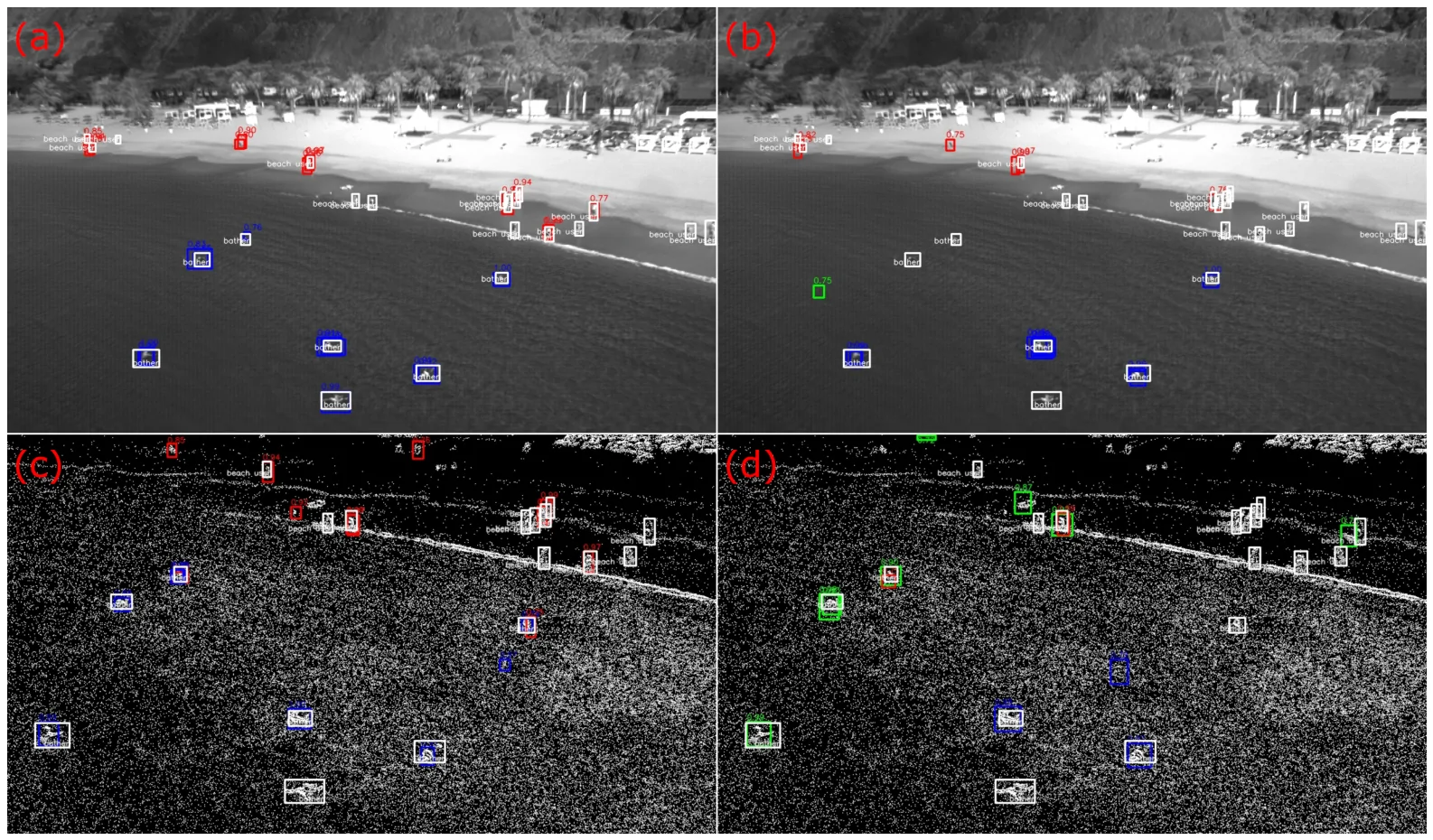
Example inference results for (**a**) YOLOv2 on frame-based images, (**b**) sYOLOv2 on frame-based images, (**c**) YOLOv2 on event data, and (**d**) sYOLOv2 on event data. Predicted beach users are marked in red, bathers in blue and surfers in green, while ground-truth bounding boxes and labels are marked in white.

**Table 1:** Dataset split: Number of samples per class (Beach User, Bather and Surfer) for each subset (training, validation and test).

Class	Training	Validation	Test
Beach User	34,647 (70%)	5726 (11%)	9454 (19%)
Bather	8776 (73%)	1630 (14%)	1553 (13%)
Surfer	8830 (72%)	1888 (15%)	1517 (12%)

**Table 2:** Network architectural complexity: Number of layers, total neurons, neurons per layer and model size in megabytes (MB).

Model	Layers	Neurons	Neuron per Layer	Model Size (MB)
YOLOv2	53	1,707,945	32,225	13.65
sYOLOv2	18	1,705,984	94,777	4.22

**Table 3:** Overall performance metrics (F1, precision, recall, mAP_50_, and mIoU) for YOLOv2, qYOLOv2, sYOLOv2, and YOLOv26 across the evaluated data modalities. Results are reported for all classes combined. YOLOv26 was evaluated using frame and event inputs. **Bold** values indicate the best result for each metric within each model across input modalities.

Model	Metric	All Classes		
		Frames	Events	Tensors
YOLOv2	F1	**0.215**	0.201	0.114
	Precision	**0.227**	0.194	0.129
	Recall	0.204	**0.209**	0.102
	mAP_50_	**0.212**	0.133	0.029
	mIoU	**0.640**	0.634	0.614
qYOLOv2	F1	0.155	**0.168**	0.080
	Precision	**0.189**	0.158	0.121
	Recall	0.131	**0.179**	0.060
	mAP_50_	0.105	**0.111**	0.020
	mIoU	0.619	**0.648**	0.613
sYOLOv2	F1	0.154	**0.172**	0.085
	Precision	**0.189**	0.161	0.124
	Recall	0.130	**0.184**	0.065
	mAP_50_	0.106	**0.114**	0.022
	mIoU	0.608	**0.647**	0.611
YOLOv26	F1	**0.323**	0.024	–
	Precision	**0.912**	0.863	–
	Recall	**0.196**	0.012	–
	mAP_50_	**0.465**	0.268	–
	mIoU	**0.712**	0.676	–

**Table 4:** Per-class performance metrics (F1, precision, recall, AP_50_, and mIoU) for YOLOv2, qYOLOv2, sYOLOv2, and YOLOv26 across the evaluated data modalities. Results are reported separately for Beach User, Bather, and Surfer. YOLOv26 was evaluated using frame and event inputs. **Bold** values indicate the best result for each metric within each model across input modalities.

Model	Metric	Beach User			Bather			Surfer		
		Frames	Events	Tensors	Frames	Events	Tensors	Frames	Events	Tensors
YOLOv2	F1	**0.204**	0.192	0.131	**0.444**	0.392	0.144	0.048	**0.074**	0.050
	Precision	**0.278**	0.190	0.167	0.314	**0.326**	0.180	0.038	**0.073**	0.042
	Recall	0.161	**0.194**	0.108	**0.754**	0.490	0.120	0.063	**0.075**	0.063
	AP_50_	0.085	**0.086**	0.040	**0.546**	0.302	0.040	0.005	**0.011**	0.006
	mIoU	**0.641**	0.637	0.617	**0.658**	0.629	0.629	0.620	**0.637**	0.595
qYOLOv2	F1	0.145	**0.210**	0.085	**0.384**	0.302	0.125	0.021	**0.077**	0.044
	Precision	0.242	**0.335**	0.160	**0.308**	0.295	0.169	0.017	**0.048**	0.042
	Recall	0.104	**0.153**	0.058	**0.507**	0.309	0.100	0.026	**0.201**	0.045
	AP_50_	0.051	**0.095**	0.019	**0.262**	0.222	0.035	0.001	**0.015**	0.006
	mIoU	0.634	**0.658**	0.618	0.616	**0.644**	0.631	0.606	**0.642**	0.590
sYOLOv2	F1	0.146	**0.217**	0.092	**0.377**	0.307	0.125	0.018	**0.079**	0.041
	Precision	0.243	**0.346**	0.161	**0.305**	0.299	0.163	0.015	**0.049**	0.041
	Recall	0.104	**0.158**	0.064	**0.495**	0.314	0.102	0.023	**0.205**	0.041
	AP_50_	0.053	**0.101**	0.023	**0.264**	0.226	0.037	0.001	**0.015**	0.005
	mIoU	0.636	**0.654**	0.610	0.614	**0.646**	0.634	0.575	**0.641**	0.590
YOLOv26	F1	**0.312**	0.002	–	**0.658**	0.172	–	**0.041**	0.001	–
	Precision	**0.916**	0.857	–	**0.904**	0.862	–	**0.889**	1.000	–
	Recall	**0.188**	0.001	–	**0.518**	0.096	–	**0.021**	0.001	–
	AP_50_	**0.415**	0.282	–	**0.771**	0.411	–	**0.208**	0.110	–
	mIoU	**0.758**	0.698	–	**0.736**	0.689	–	**0.643**	0.642	–

**Table 5:** Overall detection performance for YOLOv2, qYOLOv2, qYOLOv2-QAT, sYOLOv2, and sYOLOv2-QAT using frame, event, and tensor inputs. Results are reported over all classes combined.

Model	Input	F1	Precision	Recall	mAP_50_	mIoU
YOLOv2	Frames	0.215	0.227	0.204	0.212	0.640
	Events	0.201	0.194	0.209	0.133	0.634
	Tensors	0.114	0.129	0.102	0.029	0.614
qYOLOv2	Frames	0.155	0.189	0.131	0.105	0.619
	Events	0.168	0.158	0.179	0.111	0.648
	Tensors	0.080	0.121	0.060	0.020	0.613
qYOLOv2-QAT	Frames	0.135	0.130	0.140	0.082	0.618
	Events	0.202	0.210	0.195	0.153	0.632
	Tensors	0.077	0.074	0.081	0.016	0.610 *
sYOLOv2	Frames	0.154	0.189	0.130	0.106	0.608
	Events	0.172	0.161	0.184	0.114	0.647
	Tensors	0.085	0.124	0.065	0.022	0.611
sYOLOv2-QAT	Frames	0.135	0.130	0.141	0.080	0.617
	Events	0.205	0.214	0.197	0.151	0.636
	Tensors	0.078	0.074	0.082	0.017	0.612 *

* For tensor-based qYOLOv2-QAT and sYOLOv2-QAT, no true-positive Surfer detections were obtained at the selected confidence threshold of 0.7; therefore, overall mIoU was calculated using the Beach User and Bather class-wise mIoU values only.

**Table 6:** Backend timing and throughput results for each model, platform, and input representation. Values are reported as mean ± standard deviation over five runs.

Model/Platform	Input	Preprocessing Time (ms)	Postprocessing Time (ms)	Backend Execution Time (ms/Full-Resolution Sample)	Backend Throughput (Full-Resolution Samples/s)	Wake-Up Time (s)
YOLOv2–CPU	Frames	20.412±0.026	116.759±1.059	626.973±2.762	1.595±0.007	48.394±3.988
	Events	10.848±0.048	132.684±1.842	629.663±4.747	1.588±0.012	49.590±2.787
	Tensors	74.576±0.373	109.039±2.017	725.509±2.639	1.378±0.005	60.550±2.651
qYOLOv2–CPU	Frames	20.936±0.156	96.915±1.978	994.749±52.555	1.007±0.050	62.841±1.421
	Events	11.120±0.086	183.476±3.116	973.933±2.930	1.027±0.003	65.854±1.631
	Tensors	77.467±0.149	80.792±1.372	1089.095±12.036	0.918±0.010	64.596±1.662
sYOLOv2–CPU	Frames	20.567±0.350	91.678±0.447	1460.904±2.420	0.685±0.001	8.718±3.277
	Events	11.237±0.162	142.290±2.292	1421.130±1.256	0.704±0.001	7.622±0.128
	Tensors	76.119±0.400	78.942±1.374	1606.769±1.246	0.622±0.000	24.380±1.773
YOLOv2–GPU	Frames	22.300±0.169	269.292±2.118	205.667±0.661	4.862±0.016	70.468±4.456
	Events	11.496±0.094	302.189±8.207	206.349±1.732	4.846±0.040	70.126±2.688
	Tensors	84.857±1.003	256.837±2.476	293.075±3.738	3.413±0.043	83.768±4.564
qYOLOv2–GPU	Frames	22.814±0.493	205.648±2.851	288.988±11.753	3.465±0.140	91.540±3.021
	Events	12.561±0.706	405.129±3.378	324.931±5.575	3.078±0.054	89.956±0.790
	Tensors	84.212±0.535	176.020±1.370	373.575±4.095	2.677±0.029	93.850±3.947
sYOLOv2–AKD1000	Frames	41.536±0.409	351.094±1.026	1549.070±0.122	0.646±0.000	12.838±4.454
	Events	35.084±0.783	466.904±2.232	1643.912±0.253	0.608±0.000	9.576±2.737
	Tensors	129.934±1.323	325.753±3.603	2443.614±37.661	0.409±0.006	25.103±1.324

**Table 7:** End-to-end processing efficiency for each model, platform, and input representation. Values are reported as mean ± standard deviation over five runs. For AKD1000 configurations, end-to-end energy includes both Orin Nano host energy and AKD1000 chip energy. Orin Nano power is measured over the complete processing path, whereas AKD1000 power corresponds to backend execution.

Model/Platform	Input	End-to-End Throughput (Full-Resolution Samples/s)	End-to-End Energy (mJ/Full-Resolution Sample)	Average Orin Nano End-to-End Power (mW)	Average AKD1000 Backend Power (mW)
YOLOv2–CPU	Frames	1.308±0.005	4707.204±339.383	6158.266±428.395	–
	Events	1.293±0.009	4677.611±58.553	6048.595±46.711	–
	Tensors	1.100±0.004	5562.375±307.332	6118.353±359.865	–
qYOLOv2–CPU	Frames	0.900±0.041	10,311.123 ±527.966	9265.434±27.862	–
	Events	0.856±0.002	10,556.516 ±20.853	9032.787±6.470	–
	Tensors	0.802±0.007	11,244.516 ±106.226	9013.569±10.946	–
sYOLOv2–CPU	Frames	0.636±0.001	9016.226±1455.741	5729.767±914.678	–
	Events	0.635±0.001	9512.322±861.843	6040.200±543.089	–
	Tensors	0.568±0.001	10,863.365 ±799.006	6165.801±457.463	–
YOLOv2–GPU	Frames	2.011±0.010	2886.551±107.614	5804.001±213.177	–
	Events	1.923±0.025	3117.238±111.977	5993.002±182.519	–
	Tensors	1.575±0.014	3982.227±345.972	6271.654±525.043	–
qYOLOv2–GPU	Frames	1.934±0.054	5176.707±70.405	10,006.183 ±144.195	–
	Events	1.347±0.015	6378.905±34.704	8589.331±61.430	–
	Tensors	1.578±0.013	5976.903±176.629	9429.796±298.216	–
sYOLOv2–AKD1000	Frames	0.514±0.000	14,596.105 ±1143.474	6436.271±589.343	1348.352±0.332
	Events	0.466±0.000	15,019.201 ±168.306	5954.968±82.661	1357.153±0.558
	Tensors	0.345±0.005	20,845.101 ±1123.312	6215.594±265.702	1223.615±4.399

**Table 8:** Acquisition system current and power consumption for frame- (Blackfly S) and event-based (EVK4-HD) cameras. Power was calculated using the nominal USB supply voltage of 5.00 V.

		Blackfly S	EVK4-HD
**Stand-by**	Current	512.41 mA	89.62 mA
	Power consumption	2.56 W	0.45 W
**Running**	Current	560.02 mA	99.05 mA
	Power consumption	2.80 W	0.50 W

**Table 9:** sYOLOv2 model sparsity for frames, events, and tensor data.

Frames	Event	Tensors
0.609	0.600	0.596

## Data Availability

The dataset and the code are proprietary to ARQUIMEA and, due to contractual restrictions, the authors are not allowed to publish the full dataset or the code. To enable independent verification of the evaluation data used in the study, the test split (frames, events, and tensors) with annotations has been deposited on Zenodo: https://doi.org/10.5281/zenodo.17830310. The training and validation splits remain internal to ARQUIMEA and may be made available on a case-by-case basis following a justified request and subject to the applicable contractual restrictions. The YOLOv2-derived models employed in this study use an AkidaNet backbone. A compatible YOLOv2 starting architecture can be instantiated from the BrainChip Akida model library (https://brainchip-inc.github.io/akida_examples_2.10.0-doc-1/api_reference/akida_models_apis.html#akida_models.yolo_base (accessed on 27 July 2026)), and subsequently configured according to the network architecture and training procedure described in this manuscript. This architecture provides a compatible starting point for quantization and subsequent conversion and deployment on the AKD1000 through the Akida V1/CNN2SNN workflow. The YOLOv26 implementation used is publicly available through the Ultralytics framework (https://docs.ultralytics.com/models/yolo26/ (accessed on 27 July 2026)).

## Supplementary Materials

The following supporting information can be downloaded at https://www.mdpi.com/article/10.3390/s26217034/s1, Figure S1: Boxplots showing the timing measurements for each model (YOLOv2, qYOLOv2, sYOLOv2), computation platform (CPU, GPU, AKD1000) and data type (Frames, Events, Tensors) combination: (a) Preprocessing time (ms), (b) postprocessing time (ms), (c) backend execution time (ms/full-resolution sample), and (d) wake-up time (s). Each box displays the median (central line), the interquartile range (box limits), whiskers extending to 1.5 times the interquartile range, and outliers plotted individually; Figure S2: Boxplots showing the efficiency measurements for each model (YOLOv2, qYOLOv2, sYOLOv2), computation platform (CPU, GPU, AKD1000) and data type (Frames, Events, Tensors) combination: (a) End-to-end throughput (full-resolution samples/s), (b) end-to-end energy (mJ/full-resolution sample), (c) average Orin Nano end-to-end power (mW), and (d) average AKD1000 backend power (mW). Each box displays the median (central line), the interquartile range (box limits), whiskers extending to 1.5 times the interquartile range, and outliers plotted individually.
