# Supplementary material for: UAV-Based Person Detection with Frame and Event Sensors: A Model–Platform Case Study on Jetson Orin Nano and BrainChip AKD1000 Neuromorphic Hardware
Source: Sensors (Basel). 2026 Nov 6;26(21):7034. doi: 10.3390/s26217034 (PMC13766520; doi:10.3390/s26217034)
Supplement: Supplementary file 1 [file sensors-26-07034-s001.zip › sensors-4497938-supplementary.pdf]

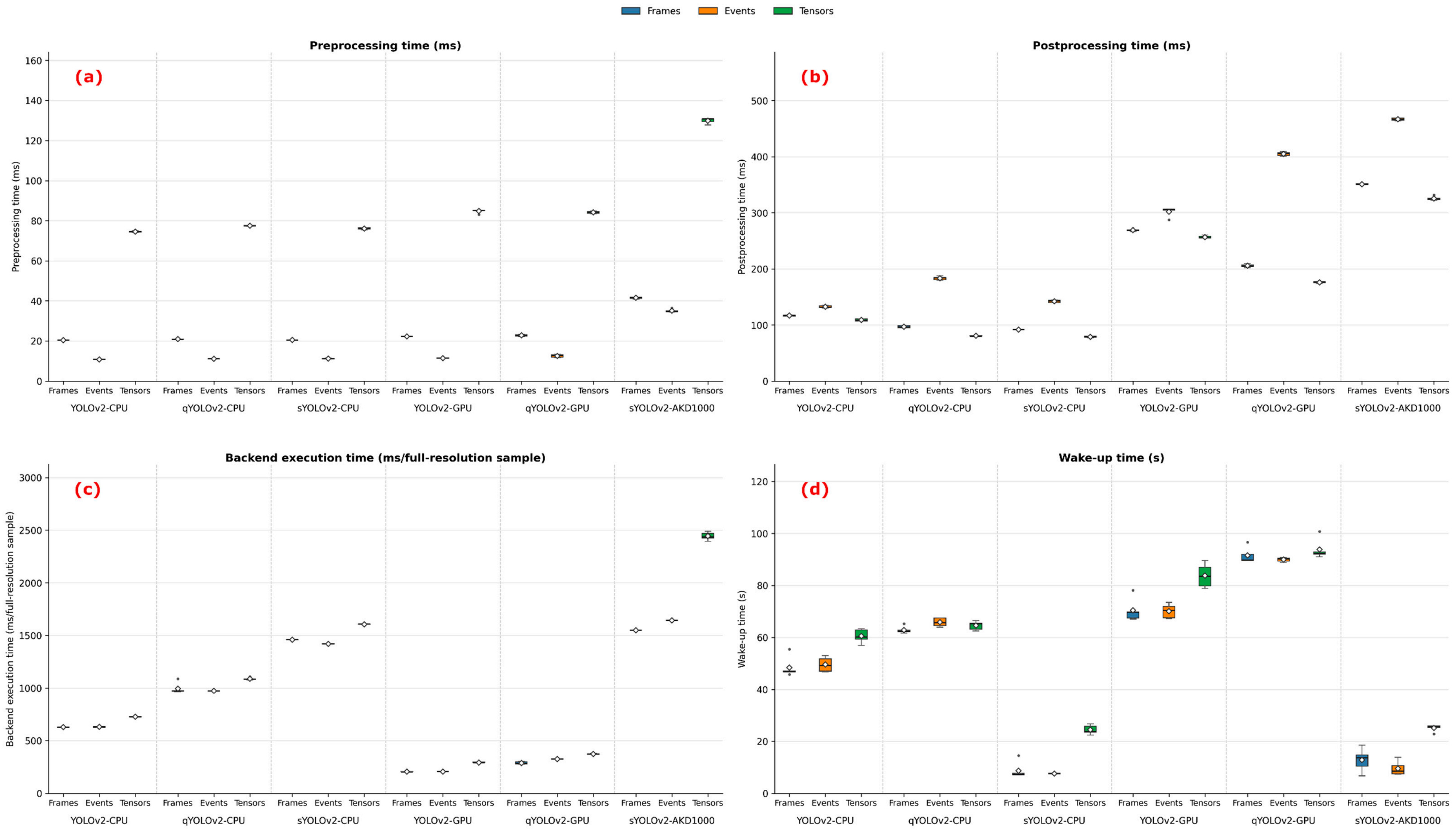

Figure S1: Boxplots showing the timing measurements for each model (YOLOv2, qYOLOv2, sYOLOv2), computation platform (CPU, GPU, AKD1000) and datatype (Frames, Events, Tensors) combination: (a) Preprocessing time (ms), (b) Postprocessing time (ms), (c) Backend execution time (ms/full-resolution sample), and (d) Wake-up time (s). Each box displays the median (central line), the interquartile range (box limits), whiskers extending to 1.5 times the interquartile range, and outliers plotted individually.

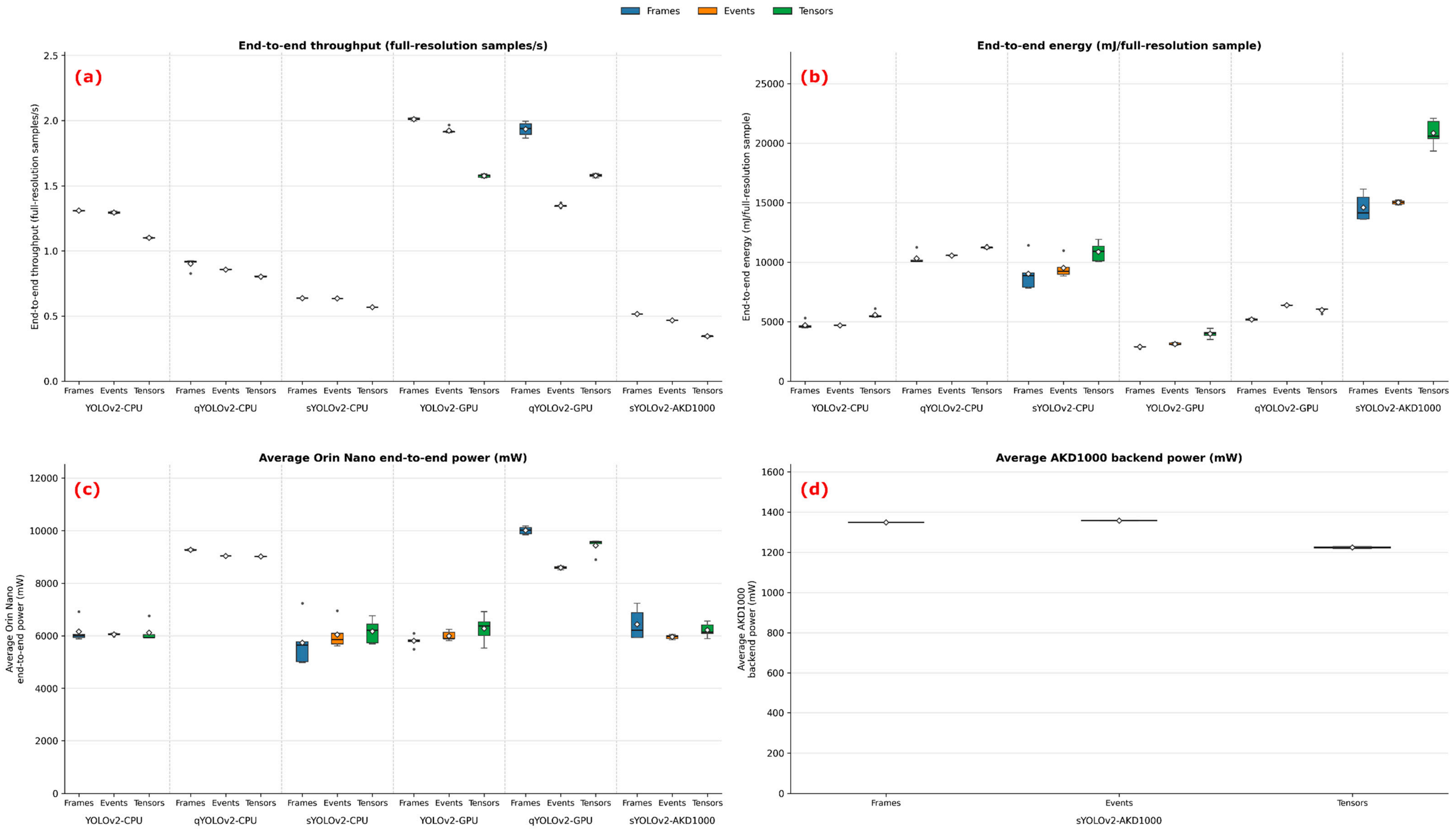

Figure S2: Boxplots showing the efficiency measurements for each model (YOLOv2, qYOLOv2, sYOLOv2), computation platform (CPU, GPU, AKD1000) and datatype (Frames, Events, Tensors) combination: (a) End-to-end throughput (full-resolution samples/s), (b) End-to-end energy (mJ/full-resolution sample), (c) Average Orin Nano end-to-end power (mW), and (d) Average AKD1000 backend power (mW). Each box displays the median (central line), the interquartile range (box limits), whiskers extending to 1.5 times the interquartile range, and outliers plotted individually.
